# Polysaccharide of *Atractylodes macrocephala* Koidz (PAMK) Alleviates Cyclophosphamide-induced Immunosuppression in Mice by Upregulating CD28/IP3R/PLCγ-1/AP-1/NFAT Signal Pathway

**DOI:** 10.3389/fphar.2020.529657

**Published:** 2020-12-08

**Authors:** Xuelian Xiang, Nan Cao, Feiyue Chen, Long Qian, Yifei Wang, Yunmao Huang, Yunbo Tian, Danning Xu, Wanyan Li

**Affiliations:** Guangdong Province Key Laboratory of Waterfowl Healthy Breeding, College of Animal Science and Technology, Zhongkai University of Agriculture and Engineering, Guangzhou, China

**Keywords:** CD28 signaling pathway, Th1/Th2, lymphocyte activation, CTX, PAMK

## Abstract

The polysaccharide of *Atractylodes macrocephala* Koidz (PAMK) is recognized as an immune enhancer, with anti-cancer, anti-tumour, lymphocyte-activating and lymphocytes proliferation-inducing effects. For investigating the mechanism that PAMK alleviates the decline in T cell activation induced by CTX, 24 6-week-old BALB/c female mice were randomly divided into four groups (C, PAMK, CTX, PAMK + CTX). The spleen index, splenocytes morphology and death, cytokine concentration, T cell activating factors (CD25, CD69, CD71), mRNA expression levels related to the CD28 signal pathway were detected. Furthermore, the lymphocytes of mice was isolated and cultured, and then the Th1/Th2 ratio, activating factors, mRNA levels related to the CD28 signal pathway were detected. The results showed that PAMK significantly improved the spleen index, alleviated abnormal splenocytes morphology and death, maintained the balance of Th1/Th2 cells, increased the levels of IL-2, IL-6, TNF-α, and IFN-γ, and increased the mRNA levels of CD28, PLCγ-1, IP3R, NFAT, and AP-1. In conclusion, PAMK increased cytokines levels and alleviated the decline in activation level of lymphocytes induced by CTX through CD28/IP3R/PLCγ-1/AP-1/NFAT signal pathway.

## Introduction

The polysaccharide of *Atractylodes macrocephala* Koidz (PAMK), the main active ingredient of *Atractylodes macrocephala*, has been confirmed to possess anti-oxidative, anti-inflammatory, anti-tumour, and anti-cancer properties, to relieve heat stress and to improve immunity (Xu and Tian, 2015; Guo et al., 2019). Regarding immunity, PAMK can significantly improve index of spleen and thymus and spleen lymphocytes proliferation, promote T lymphocytes to enter S and G2/M phases, and increase T/B cell proportions, NK cytotoxicity, cytokine levels (IL-4, IL-5, IL-6, IL-10, TNF-α, IFN-γ), and serum specific immunoglobulin G (IgG) titers, thereby improving immune function (Li et al., 2009; Guo et al., 2012b; Liu et al., 2015; Xu et al., 2019). In addition, Xu *et al* also found that PAMK alleviated heat stress by decreasing levels of TNF-α, IFN-γ, IL-4, heat shock protein 60 (HSP60), and heat shock protein 70 (HSP70) in chicken (Xu and Tian, 2015). However, the mechanism that PAMK enhances cytokines and activates lymphocytes is still unknown. A novel polysaccharide obtained from *Craterellus cornucopioides* has been reported to activate spleen lymphocytes in mice through the TLR4-MyD88-NF-κB pathway (Guo et al., 2019), while *Ganoderma lucidum* polysaccharides might induce activation of spleen lymphocytes via the Ca^2+^/CaN/NFAT/IL-2 signal pathway (Yu et al., 2015). Thus, exploring the signaling pathway that PAMK alleviates the immunosuppression in mice induced by cyclophosphamide (CTX) would be exciting research interest.

CD28 is an important signal pathway geen for T lymphocyte activation. As an important co-stimulatory signaling molecule, CD28 can enhance the secretion of IL-2 and other cytokines, upregulating the expression of cell survival genes, and promote cell division and energy metabolism. CD28 is an important costimulatory lymphoid molecule required for full activation of T lymphocytes and plays an important role in T cell activation (Zhang et al., 2017). CD28 binds to CD86 and CD80 receptors on antigen presenting cells (APC), mediates T cell co-stimulation, promotes proliferation and differentiation and produces a range of cytokines. CD28 receptor cross-linking induces phospholipase C gamma 1 (PLCγ-1) phosphorylation, and then PLCγ-1 hydrolyses inositol phospholipid on the membrane to produce inositol 1,4,5-triphate receptor (IP3R) and DAG. DAG activates the protein kinase C (PKC) signal pathway, resulting in the phosphorylation of proteins such as nuclear factor of activated T cells (NFAT), activator protein 1 (AP-1) and nuclear factor kappa-B (NF-κB) (Ding et al., 2019a). CD28 pathway activation can activate genes that encode cytokines such as IL-2, IL-2R, IL-4, IL-6, and IFN-γ, leading to T cell activation, entry into the cell cycle and promotion of cytokines secretion to enhance the immune response.

To investigate the mechanism that PAMK alleviates lymphocyte damage induced by CTX, immunosuppressive mice model was constructed, and the CD28 signal pathway, which is closely related to lymphocytes activation, was chosen to investigate the effects of PAMK on the spleen organ index, spleen cell morphology and function, cell cytokines (IL-2, IL-6, TNF-α, IFN-γ), and mRNA levels related to the CD28 signal pathway in the spleen and lymphocytes of immunosuppressive mice. This will give a preliminary concusion that how PAMK alleviates CTX -induced T lymphocyte activation in mice through upregulating CD28 and downstream signal pathway.

## Material and Methods

### Experiment Grouping and Treatments

Specific pathogen free (SPF) mice were purchased from the Traditional Chinese Medicine University Of Guangzhou. The mice were housed in a specific pathogen-free environment (12/12- h light/dark cycle, 22–24°C, 40–60% humidity) and randomly divided into four groups (C, CTX, PAMK, CTX + PAMK; six mice/group). All the mice were treated humanely, and the experiments received prior ethical approval in accordance with Zhongkai University of Agriculture and Engineering under the approved protocol number SRM-11.

PAMK (purity 95%) was purchased from Yanglingciyuan Biotechnology company (Xi’an, China). The four groups of mice had free access to food and water. The PAMK and PAMK + CTX groups were treated with PAMK at a dose of 200 mg/(kg body weight), per day, via an oral gavage. At the same time, the C and CTX groups received the same volume of ddH_2_O. At day 25, 26, 27, the CTX and PAMK + CTX groups were treated with CTX at a dose of 100 mg/(kg body weight) by intraperitoneal injection (Figure 1). The C and PAMK groups received the same volume of saline. All the mice continue to receive the ddH_2_O and PAMK respectively until day 35. At day 35, the spleen was collected and weighed; all the serum and samples were stored at −80°C.

**FiIGURE 1:**
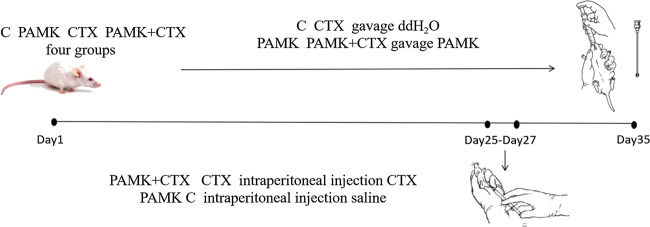
Schematic outlines of the experimental approaches tested in mice.

### The Spleen Index Assay

The weight of the spleens was measured immediately after the mice were euthanized. The spleen index was the ratio of spleen weight (g) to body weight (kg).

### Spleen Histology and Ultramicroscopic Morphology Observation

HE staining: Paraffin-fixed blocks were serially cut into 5-6 μm-thick coronal sections. For routine histological examination, the paraffin sections were stained with HE. HE-stained slices were analyzed under a Nikon upright microscope (Nikon Eclipseci, Tokyo, Japan). 10 fields of view (100 ×) were randomly selected from each group to measure the thickness of the periarterial lymphatic sheath in each field (Mingmei Optical Fiber Digital Measurement and Analysis System V1.5.3) for statistical analysis. Three slices were selected from each group and 15 fields (400 ×) were randomly captured to calculate the number of megakaryocyte precursor cells in each field of view, and performe statistical analysis.

TEM: Each spleen was divided into small blocks of 1 mm three and then fixed with 2.5% glutaraldehyde at 4°C. Ultrathin slices with a thickness of 50–70 nm were prepared and stained with uranyl acetate (22,400, EMS, USA) 30 min and lead citrate (19,314, TED PELLA, USA) 15 min The samples were examined under a transmission electron microscope (JEM-1400, Tokyo, Japan). 20 fields of view (2,500 ×) were randomly selected from each group to calculate the total number of cells, the number of apoptotic cells in each field, and proportion of cell apoptosis, then performe statistical analysis.

### TUNEL Assay for Death of Spleen Cells

The blank portions of the HE-stained sections were stained according to the TUNEL kit (11684,817,910, Roche, USA) instructions and analyzed under a Nikon upright microscope (Nikon Eclipseci, Tokyo, Japan). Two sections from each mouse were randomly selected from five horizons magnified at ×400. Each field of vision was analyzed to obtain the positive cell number and the total number of cells to calculate the percentage of positive cells. All the results was analyzed by ImageJ (National Institutes of Health,MD, USA).

### Quantitative Reserve-Transcription PCR Analysis

Total RNA was extracted from the spleen with TRIzol reagent (Thermo Fisher, USA) and reverse-transcribed was performed by the Reverse Transcription Kit (Takara, Japan). The primers (Table 1) of related genes were designed by using Primer Premier 5.0 software (Premier Biosoft International, USA). An ABI PRISM 7500 detection system (Applied Biosystems, Foster City, CA) was used to measure the mRNA levels of genes related to the CD28 signal pathway.

**TABLE 1:** Primer sequences for qPCR.

cDNA	Primer sequences	cDNA	Primer sequences
TNFα-F	GTTCTATGACCGCCCAGTTC	IFN-γ-F	AGATGTAGCTGACGGTGGAC
TNFα-R	CACACAGACAGCCAAGTCAA	IFN-γ-R	ATGTGTTTGATGTGCGGCTT
CD28-F	ACAACGAGAGGAGCAATGGA	IP3R-F	AAGGAGGTGATGTGGTGAGG
CD28-R	GCTGGTAAGGCTTTCGAGTG	IP3R-R	GCCAAGTAATGCCCTGTAGC
IL-6-F	AGACTTCCATCCAGTTGCCT	PLCγ1-F	AGATGAACCAGGCCCTCTTC
IL-6-R	CATTTCCACGATTTCCCAGAG	PLCγ1-R	AGCCACCTCAATCTCCACAA
IL-2-F	AGCAGCTGTTGATGGACCTA	NFAT-F	TTGAGCTGAGGAAAGGGGAG
IL-2-R	GGTCTCAGTTGGTGTGTAGAG	NFAT-R	TGACTGGGTAGCTGTCTGTG
CD71-F	TGGACATGCTCATCTAGGAA	GAPDH-F	GGCCTCCAAGGAGTAAGAAA
CD71-R	ATTAGGCAACCCTGATGACT	GAPDH-R	GCCCCTCCTGTTATTATGG

### Assays of Cell Cytokines in Serum

The blood of mice was collected and centrifuged to separate serum. The levels of IL-2 (XY-ELA9021), IL-6 (XY-ELA0018), TNF-α(XY-ELA0023), and IFN-γ(XY-ELA0026) were measured by using enzyme-linked immunosorbent assay (ELISA) kits (Shanghai Xinyu Technology Company, Shanghai, China).

### Culture and Transfection of Spleen Lymphocytes

The mouse spleens were aseptically extracted and separated into single lymphocytes with lymphocyte separation solution (MPbio, USA), adjusted to a density of 3×10^6^ cells/mL, and cultured in a 12-well plate (Corning Incorporated, USA). After 4 h of culture, transfection reagent siCD28 (RiboBio Co., LTD., Guangzhou, China) (Table 2) was added, and cells were collected 24 h later for mRNA expression detection.

**TABLE 2:** The sequence of siCD28.

Name	Target sequences	Name	Target sequences
SiCD28–1	GGCTCTCAACTTCTTCTCA	SiCD28–3	TCACTCGAAAGCCTTACCA
SiCD28–2	GGAATCTGCACGTCAATCA	—	—

### Flow Cytometry

Mouse spleen was dissected aseptically, and then lymphocytes were isolated in lymphocyte separation fluid. We incubated mouse lymphocytes at 37°C in a 5% CO_2_ incubator for 4 h followed by centrifugation at 2000 rpm for 5 min and collection. After sufficient mixing with 1 ml of PBS, we centrifugated it at 2000 rpm for 5 min and discarded the supernatant. After the lymphocytes were collected, the previous step was repeated once. The cell density was adjusted to 2 × 10^7^ cells/mL with 1 × staining buffer, and we took out 50 µL of cells to the flow cytometer tube. The cells were then completely mixed with 10 µL of anti-CD3-FITC (BD553061, BD, USA) and anti-CD4-APC (BD561091, BD, USA) for 30 min on ice and protected from light. Next, 250 μL of Fixation/Permeabilization solution was added to each tube, followed by two washes with 1×BD Perm/Wash^™^ buffer, and addition of 10 μL anti-IL-4-PE (BD554435, BD, USA) and anti-IFN-γ-Cy5.5 (BD560660, BD, USA). We collected 10,000 lymphocytes with CellQuest to calculate the Th1/Th2 cell ratios and then analyzed them in Flowjo (BD C6, USA).

### Statistical Analysis

The data were analyzed using SPSS for Windows (version 18, SPSS Inc., Chicago, IL, USA) and expressed as the means ± standard deviations (SD). All the qPCR assays were repeated in triplicate, and the relative expression levels were measured in terms of threshold cycle (Ct) values and normalized via formula 2^*−∆∆Ct*^. Differences between groups were compared using one-way analysis of variance (ANOVA) followed by Tukey’s honestly significant difference test. *p* < 0.05 was considered statistically significant.

## Results

### Polysaccharide of *Atractylodes macrocephala* Koidz Alleviates Cyclophosphamide -Induced Spleen Damage

**FIGURE 2:**
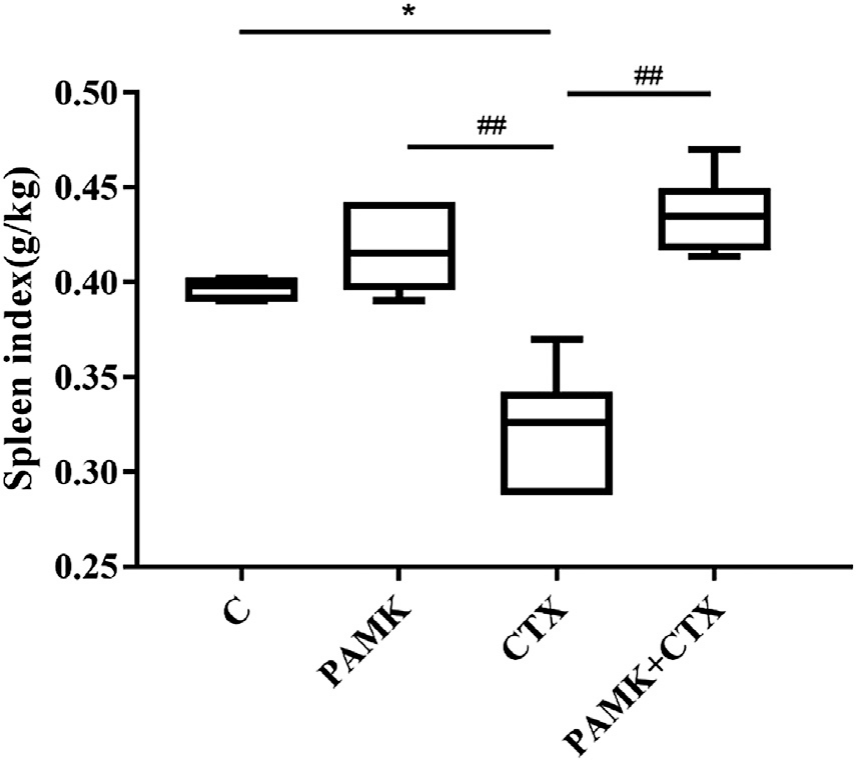
Effects of PAMK on the spleen index decrease induced by CTX. The data are expressed as the means ± SD, n = 6. *p < 0.05, **p < 0.01, ***p < 0.001, compared with C group; ^#^ *p* < 0.05, ^##^ *p* < 0.01, ^###^ *p* < 0.001, compared with CTX group.

As presented in Figure 2, the results showed that CTX could reduce the spleen index. While PAMK increased the spleen index significantly compared with CTX group but showed no significant difference from C group. The spleen index of PAMK + CTX group was significantly higher than that of CTX group, which indicated that PAMK could alleviate the decrease in the spleen index induced by CTX.

**FIGURE 3:**
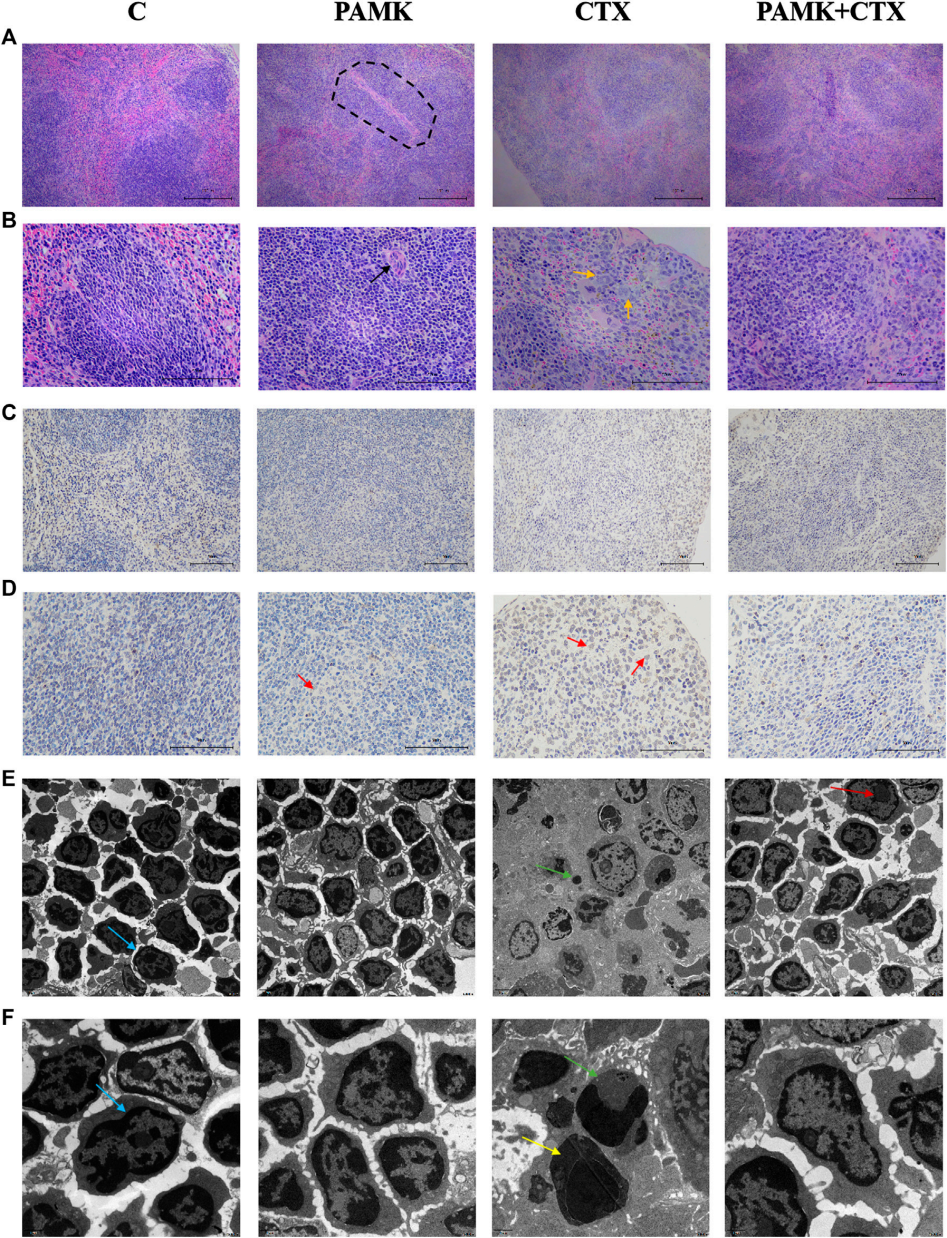
Effects of PAMK on histology, ultramicroscopic morphology and TUNEL of mouse spleen treated with CTX **(A)** HE staining of the spleen (×200); **(B)** HE staining of the spleen (×400); **(C)** TUNEL of the spleen (×200); **(D)** TUNEL of the spleen (×400); **(E)** transmission electron microscopy (TEM; ×5,000) of the spleen. **(F)** Transmission electron microscopy (TEM; ×12,000) of the spleen. The circle highlights the splenocytes sheath surrounding the arteries, the black arrow indicates the central artery, the orange arrow indicates a macrophage, the red arrow indicates dead cell nuclei stained brown, the blue arrow indicates normal splenocytes, the yellow arrow indicates splenocytes undergoing apoptosis, the green arrow indicates an apoptotic body, and the deep red arrow indicates the cell nucleus.

The results of HE staining **(** Figures 3A,B **)** showed that the red and white pith in group C was clearly demarcated and the cells were neatly arranged and compact. The cells were normal. The shape of the lymphatic sheath around the artery was normal, and the sheath wall was thick. The area of white pulp increased in the PAMK group, and the difference in cell morphology between the PAMK and C groups was not obvious. In the CTX group, the boundaries of red and white pith were not clear, no obvious germinal center was observed, the cells were loose and the lymphoid sheath wall around the artery became thinner. The cells in the PAMK + CTX group were arranged more closely than those in the CTX group, and the morphology was normal. The red and white pith boundaries were clearer. At high magnification, the cells in the C and PAMK groups were in good shape, with darker nuclei and tighter cell arrangements. However, the PAMK + CTX group had a more regular morphology and closer arrangement than the CTX group, with darker nuclei. The above results suggested that PAMK could alleviate the lymphocytes damage induced by CTX and reduce connective tissue hyperplasia.

The TEM results **(** Figures 3E,F **)** showed that the PAMK and C groups had an orderly cell arrangement, a regular cell morphology, a neat distribution of euchromatin and heterochromatin, and a complete nuclear membrane structure. In the CTX group, the number of spleen cells per unit area decreased, the cell morphology was incomplete, dead cells were observed, the chromatin was not evenly distributed, the chromatin was severely clustered, nuclear condensation occurred, and apoptotic bodies were observed. Compared with the CTX group, the PAMK + CTX group showed a more regular cell morphology, closer cell arrangement, increased cell number, significantly reduced apoptotic bodies, intact nuclear membrane and normal levels. These results indicated that PAMK alleviated the abnormal cell morphology and decreased the number of apoptotic bodies in lymphocytes induced by CTX.

**FIGURE 4:**
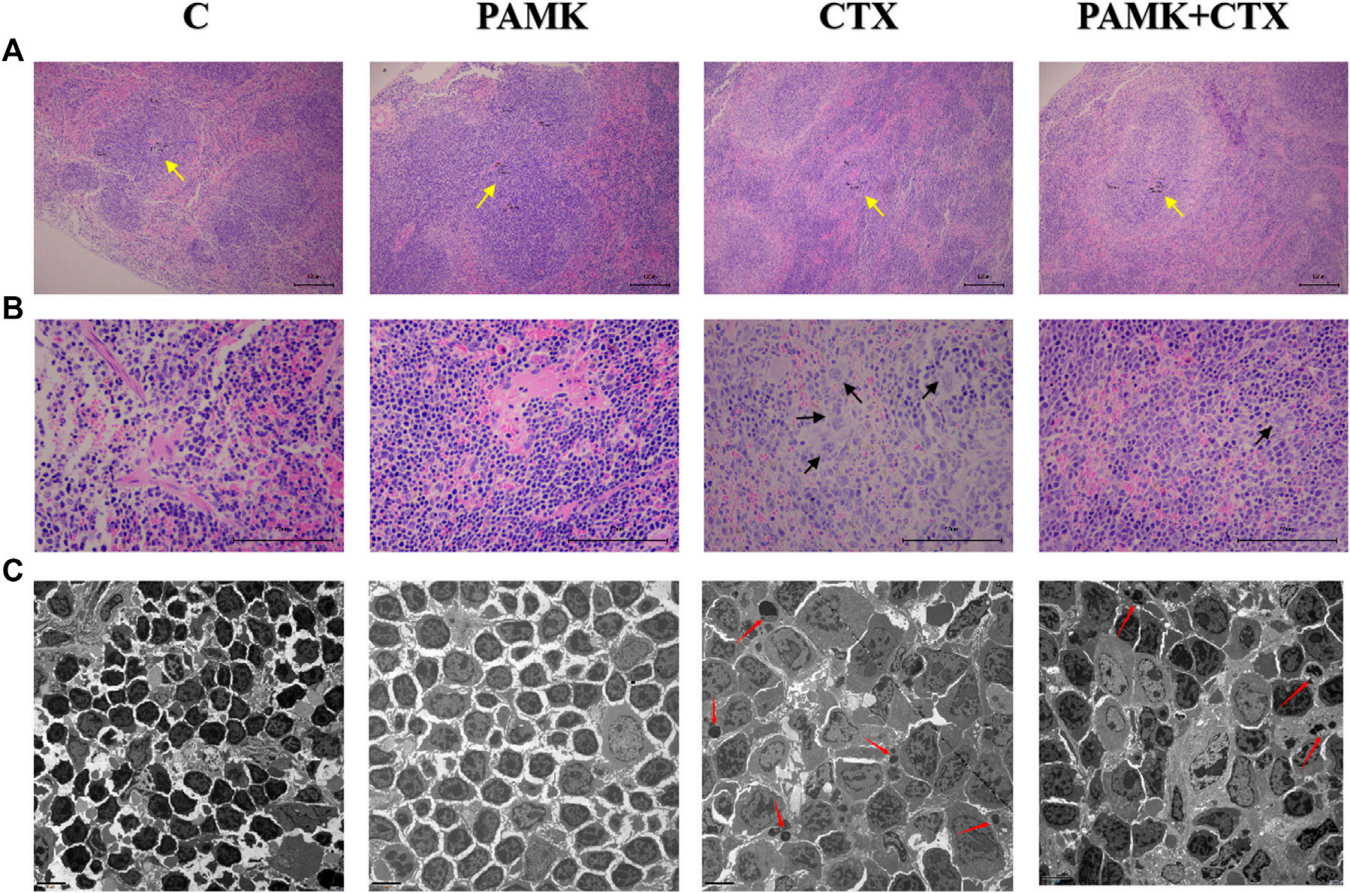
Effects of PAMK on the thickness of the periarterial lymphatic sheath, the number of megakaryocyte precursor cells and the apoptosis rate of spleen cells of mouse treated with CTX. **(A)** HE staining of the spleen (×100); **(B)** HE staining of the spleen (×400); **(C)** Transmission electron microscopy of the spleen (TEM; ×2,500). The yellow arrow indicates to the periarterial lymphatic sheath, the black arrow indicates to the megakaryocyte precursor cell, the red arrow indicates to the apoptotic body.

**FIGURE 5:**
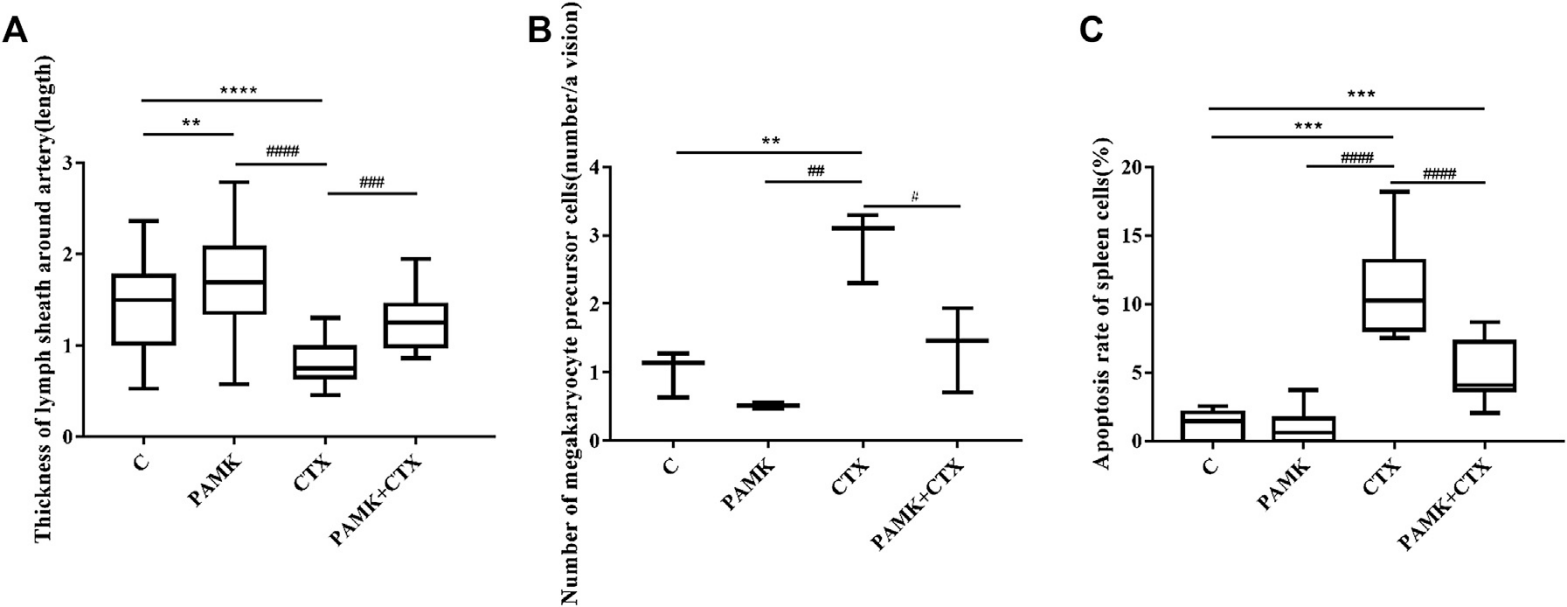
The quantification of the thickness of the periarterial lymphatic sheath, the number of megakaryocyte precursor cells and the apoptosis rate of TEM. **(A)** Thickness of periarterial lymphatic sheath (length); **(B)** Number of megakaryocyte precursor cells (number/per view); **(C)** Apoptosis rate of spleen cells (%). The data are expressed as the means ± SD. *p < 0.05, **p < 0.01, ***p < 0.001, compared with C group; ^#^ *p* < 0.05, ^##^ *p* < 0.01, ^###^ *p* < 0.001, compared with CTX group.

From Figure 4 and Figure 5, the results showed that CTX could decrease the length of the thickness of the periarterial lymphatic sheath and increase the number of megakaryocyte precursor cells and apoptotic body, but PAMK could significantly alleviate the bad affect caused by CTX, even make it back to normal level.

**FIGURE 6:**
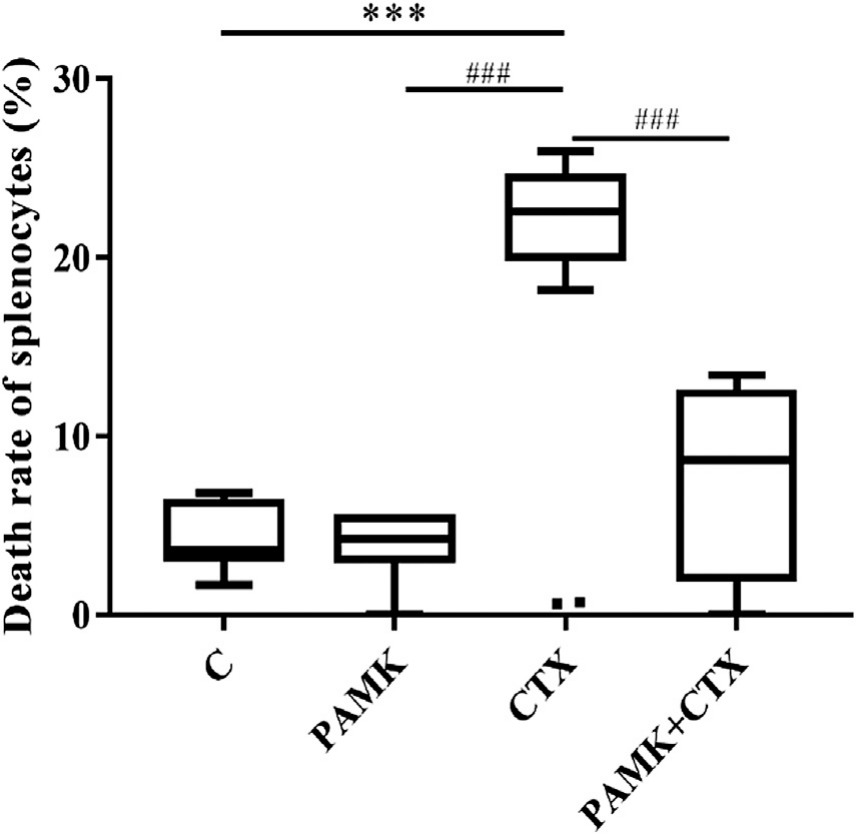
Effects of PAMK on the death rate of splenocytes of mice treated with CTX. The data are expressed as the means ± SD, n = 6.*p < 0.05, **p < 0.01, ***p < 0.001, compared with C group; ^#^ *p* < 0.05, ^##^ *p* < 0.01, ^###^ *p* < 0.001, compared with CTX group.

The TUNEL results **(** Figures 3C,D, 6 **)** showed that CTX could increase the number of dead splenocytes, while PAMK could obviously alleviate the death. The death rates of splenic splenocytes among C, PAMK, and PAMK + CTX groups were not significantly different. That results indicated that PAMK could alleviate the increased number of dead splenocytes induced by CTX.

### Polysaccharide of *Atractylodes macrocephala* Koidz Alleviates the Decrease of Cytokine Levels Induced by Cyclophosphamide

**FIGURE 7:**
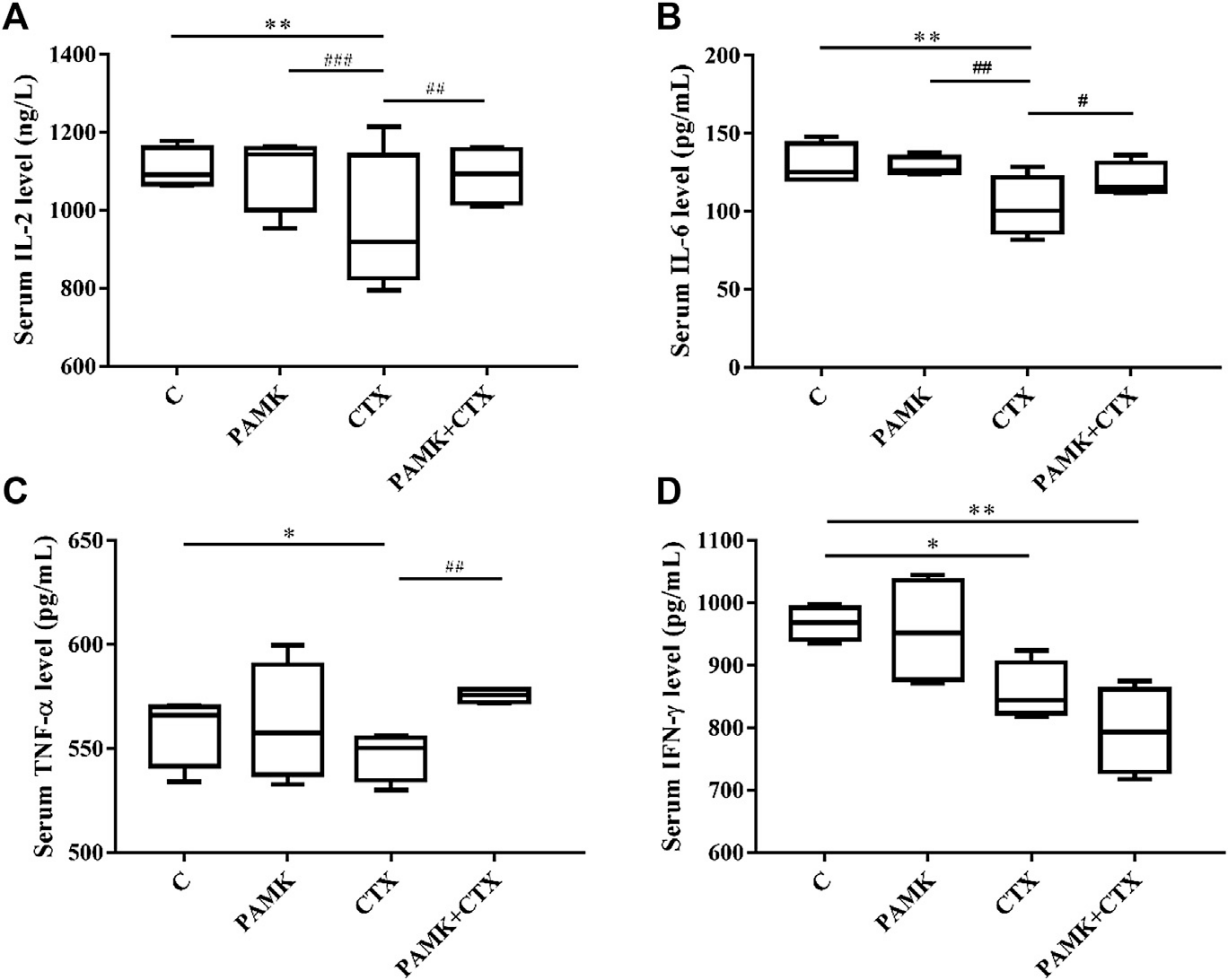
Effects of PAMK on cytokines in mice treated with CTX. Serum levels of **(A)** IL-2, **(B)** IL-6, **(C)** TNF-α, and **(D)** IFN-γ. Data are expressed as the means ± SD, n = 4.*p < 0.05, **p < 0.01, ***p < 0.001, compared with the C group; ^#^ *p* < 0.05, ^##^ *p* < 0.01, ^###^ *p* < 0.001, compared with the CTX group.

The contents of IL-2, IL-6, TNF-α, and IFN-γ in serum were measured by ELISA **(** Figure 7 **)**. Treatment with PAMK showed no significant difference compared with C group. However, CTX group showed the opposite effect: the level of IL-2, IL-6, TNF-α, and IFN-γ in serum were lower for CTX group than for C group. PAMK + CTX group significantly increased the levels of IL-2, IL-6, and TNF-α but showed an upward trend of IFN-γ compared with CTX group. When compared with C group, the levels of IL-2, IL-6, and TNF-α in PAMK + CTX group were not significantly different. PAMK could alleviate the decline in serum levels of IL-2, IL-6, TNF-α, and IFN-γ in immunosuppressed mice induced by CTX.

**FIGURE 8:**
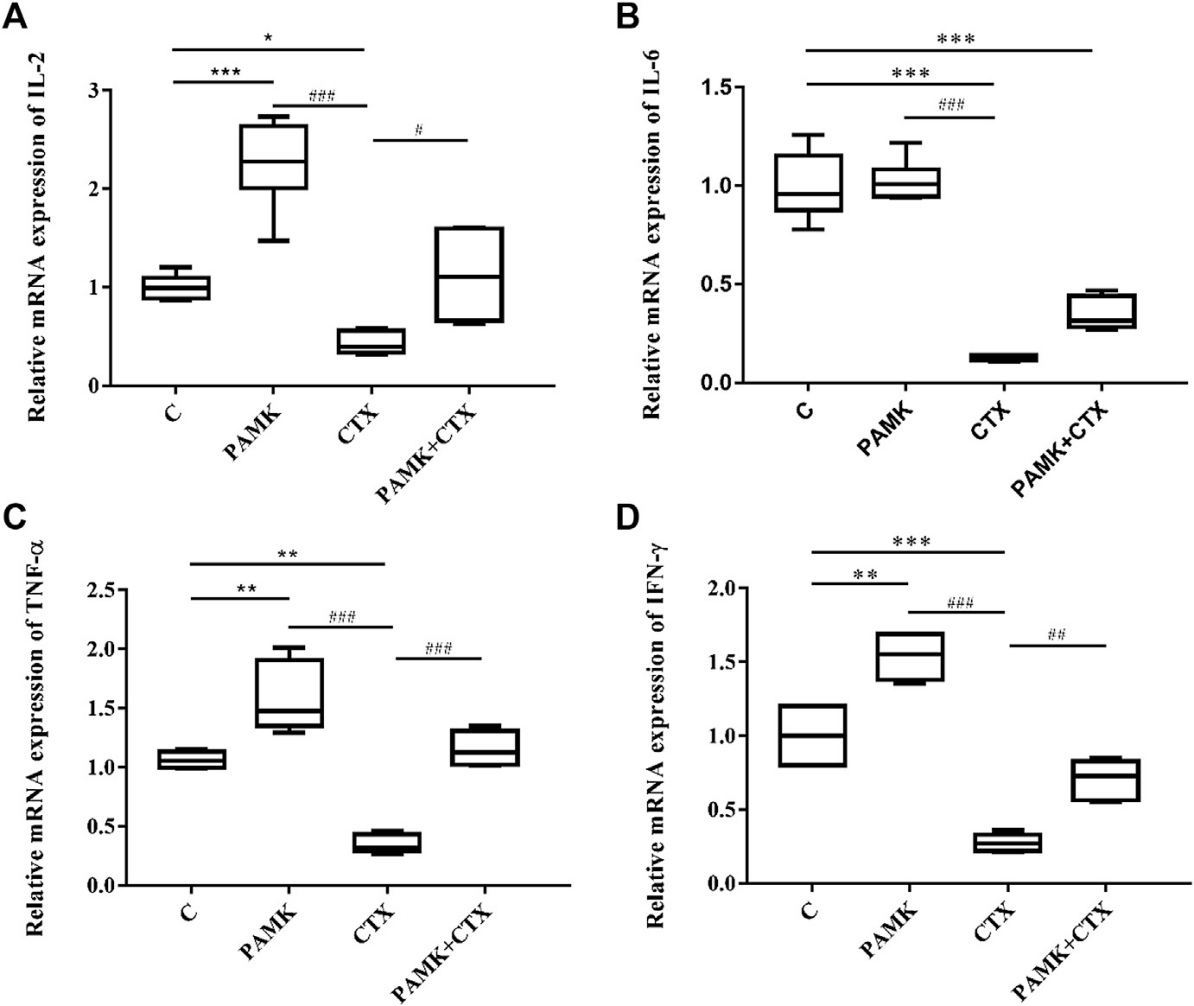
Effects of PAMK on cytokines in mice treated with CTX. Relative mRNA expression of **(A)** IL-2, **(B)** IL-6, **(C)** TNF-α, and **(D)** IFN-γ. Data are expressed as the means ± SD, n = 6. *p < 0.05, **p < 0.01, ***p < 0.001, compared with the C group; ^#^ *p* < 0.05, ^##^ *p* < 0.01, ^###^ *p* < 0.001, compared with the CTX group.

Analysis of the transcription levels of cytokines in the spleen **(** Figure 8 **)** from total RNA extracted from the spleen was performed. The results showed that the relative mRNA expression levels of IL-2, IL-6, IFN-γ and TNF-α were inhibited in CTX group. Additionally, the levels of IL-2, IFN-γ and TNF-α higher in the PAMK group than the C group, but the levels of IL-6, showed no significant difference. However, when compared with the CTX group, the relative mRNA expression of IL-2, TNF-α, IFN-γ was significantly increased in the PAMK + CTX group, with an increasing trend of IL-6 was observed. All the results showed that PAMK could alleviate the decline in the mRNA expression level of IL-2, IL-6, TNF-α and IFN-γ in immunosuppressed mice induced by CTX.

### Polysaccharide of *Atractylodes macrocephala* Koidz Alleviates the Imbalance of TH1/TH2 Induced by Cyclophosphamide

**FIGURE 9:**
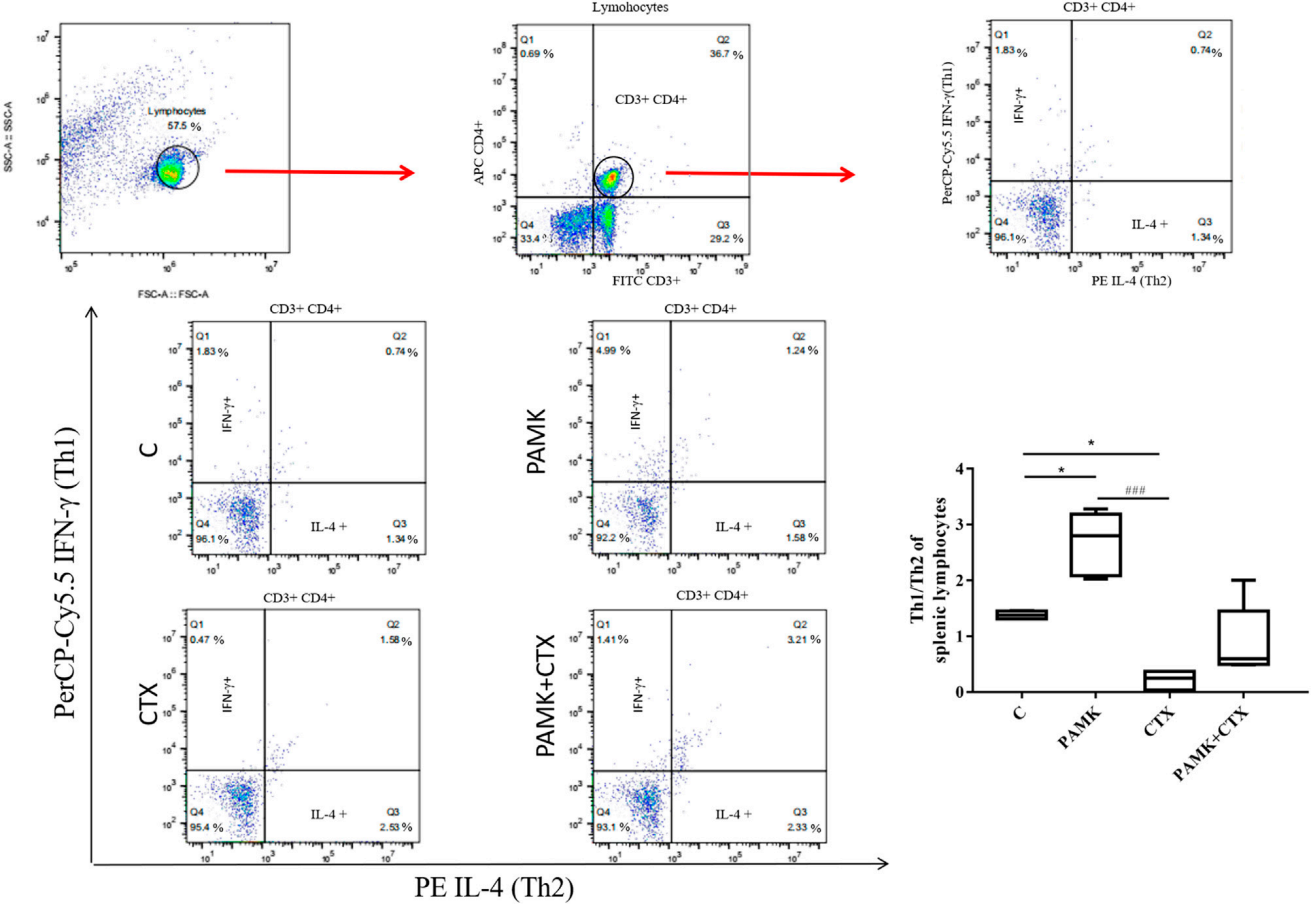
Effects of PAMK on the Th1/Th2 ratio in mice treated with CTX. Data are expressed as the means ± SD, n = 4. *p < 0.05, **p < 0.01, ***p < 0.001, compared with the C group; ^#^ *p* < 0.05, ^##^ *p* < 0.01, ^###^ *p* < 0.001, compared with the CTX group.

As shown in Figure 9, the PAMK regulated the Th1/Th2 ratio disorder in immunosuppressive mice induced by CTX. When compared with C group, the Th1/Th2 ratio in the PAMK group increased significantly, and that significantly decreased in the CTX group. In PAMK + CTX group, the Th1/Th2 ratio increased to 0.89 ± 0.28 but showed no significant difference compared with the CTX group. This result demonstrated that PAMK could alleviate the imbalance of Th1/Th2 induced by CTX.

### Polysaccharide of *Atractylodes macrocephala* Koidz Regulates mRNA Expression on CD28 and the Downstream Signal Pathway in the Spleen

**FIGURE 10:**
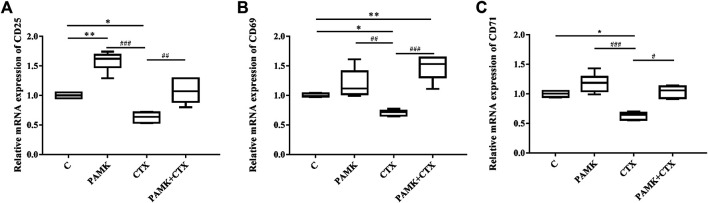
Effects of PAMK on activated factor in mice treated with CTX. Relative mRNA expression of **(A)** CD25, **(B)** CD69, and **(C)** CD71. Data are expressed as the means ± SD, n = 6. ^*^ *p* < 0.05, ^*^^*^ *p* < 0.01, ^*^^*^^*^ *p* < 0.001, compared with the C group; ^#^ *p* < 0.05, ^#^^#^ *p* < 0.01, ^#^^#^^#^ *p* < 0.001, compared with the CTX group.

We extracted total RNA from the spleen to measure relative mRNA expression levels of activated factors. Figure 10 demonstrates that CTX could decrease the relative mRNA expression levels of CD25, CD69 and CD71. For the CTX + PAMK group, PAMK was found to improve the levels of CD25 and CD71 back to normal, similar to C group. The level of CD69 even surpassed that of C group. When compared with C group, the levels of CD69 and CD71 in the PAMK group showed no significant difference with the exception of CD25. All the results suggested that PAMK could alleviate the decline in the mRNA expression level of CD25, CD69, and CD71 induced by CTX.

**FIGURE 11:**
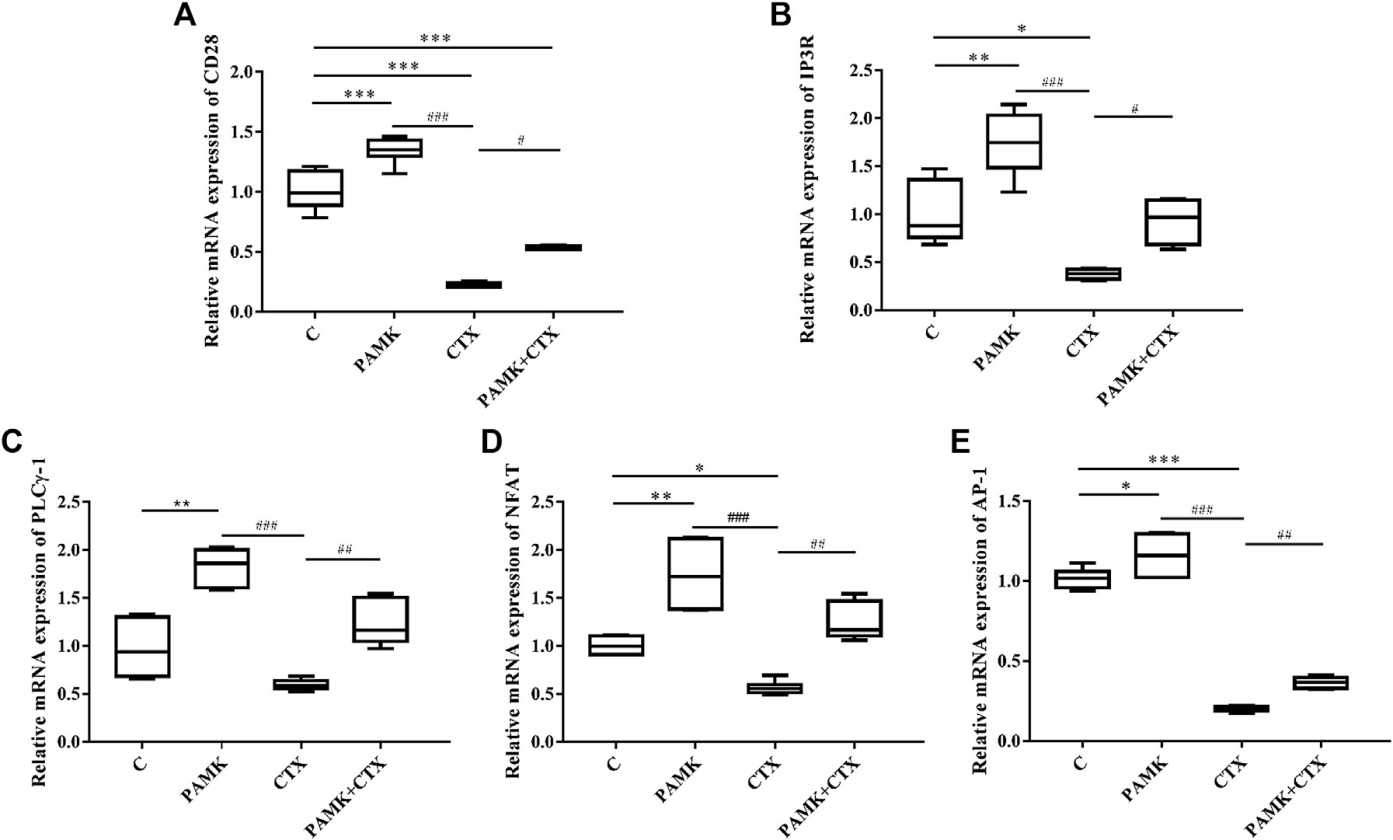
Effects of PAMK on CD28 and downstream signal pathways in mice treated with CTX. Relative mRNA expression of **(A)** CD28, **(B)** IP3R, **(C)** PLCγ-1, **(D)** NFAT, **(E)** AP-1. Data are expressed as the means ± SD, n = 6. *p < 0.05, **p < 0.01, ***p < 0.001, compared with the C group; ^#^ *p* < 0.05, ^##^ *p* < 0.01, ^###^ *p* < 0.001, compared with the CTX group.

Simultaneously, we measured the transcript mRNA levels of CD28 signaling pathway genes. As shown in Figure 11, the relative mRNA expression levels of CD28, IP3R, PLCγ-1, NFAT, and AP-1 were significantly increased after treatment with PAMK compared with C group. Additionally, CTX could significantly inhibit the relative mRNA expression of CD28, IP3R, NFAT, AP-1 and have inhibit trend with PLCγ-1. However, PAMK + CTX group significantly increased the level of CD28, IP3R, PLCγ-1, NFAT and AP-1 compared with CTX group. These results demonstrated that PAMK could alleviate the decline in the mRNA expression level of CD28, IP3R, PLCγ-1, NFAT, and AP-1 induced by CTX.

### Polysaccharide of *Atractylodes macrocephala* Koidz Regulates the mRNA Levels Expression of CD28 and the Downstream Signal Pathway Genes in Lymphocytes

**FIGURE 12:**
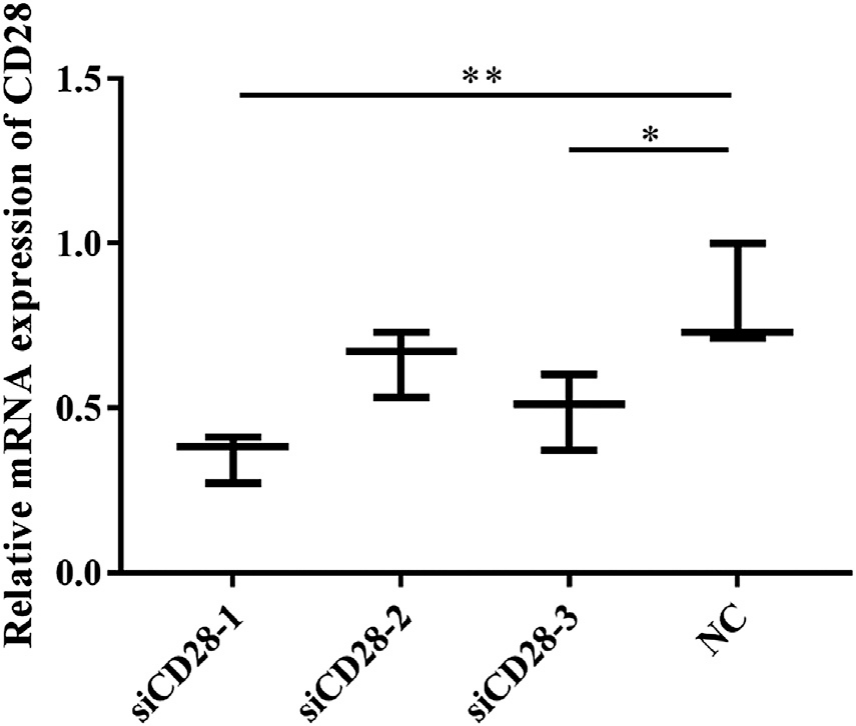
The effect of CD28-siRNA on CD28 mRNA expression. Data are expressed as the means ± SD, n = 3. NC vs all treatments: ^*^ *p* < 0.05, ^*^^*^ *p* < 0.01.

In this experiment, the optimal conditions of siCD28 to inhibit lymphocyte gene silencing were explored (Figure 12). According to some related research reports, 5 μg/ml PAMK could significantly stimulate lymphocytes proliferation. The results showed that compared with the NC group, siCD28–1 silencing efficiency was the highest at up to 60% (*p* < 0.05).

**FIGURE 13:**
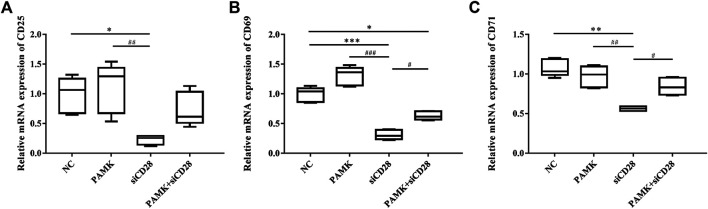
Effects of PAMK on activated factor in lymphocytes treated with siCD28. Relative mRNA expression of **(A)** CD25, **(B)** CD69, and **(C)** CD71. Data are expressed as the means ± SD, n = 6. *p < 0.05, **p < 0.01, ***p < 0.001, compared with the NC group; ^#^ *p* < 0.05, ^##^ *p* < 0.01, ^###^ *p* < 0.001, compared with the siCD28 group.

We obtained total RNA from lymphocytes to measure the relative mRNA expression levels of activated factor. Figure 13 shows that siCD28–1 could decrease the mRNA levels of CD25, CD69, and CD71. However, the combination of PAMK and siCD28 showed that PAMK improved the level of CD25, CD69 and CD71 back to normal levels close to that of NC group. These findings demonstrated that PAMK can alleviate the decline in the mRNA expression level of CD25, CD69, and CD71 induced by siCD28.

**FIGURE 14:**
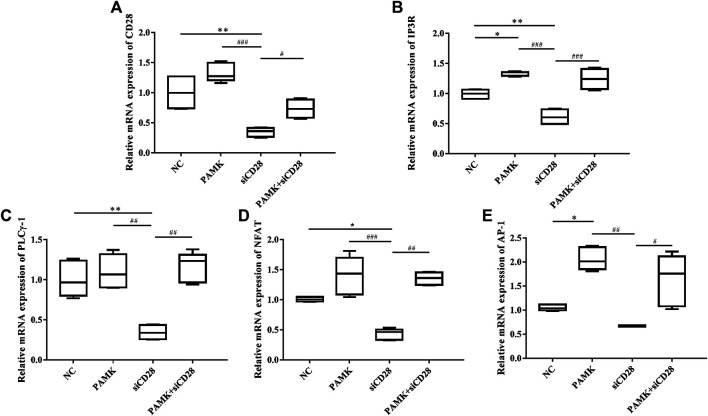
Effects of PAMK on CD28 and downstream signal pathway in lymphocytes treated with siCD28. Relative mRNA expression of **(A)** CD28, **(B)** IP3R, **(C)** PLCγ-1, **(D)** NFAT, and **(E)** AP-1. Data are expressed as the means ± SD, n = 6.*p < 0.05, **p < 0.01, ***p < 0.001, compared with the NC group; ^#^ *p* < 0.05, ^##^ *p* < 0.01, ^###^ *p* < 0.001, compared with the siCD28 group.

Next, we measured the relative mRNA expression levels of CD28 signal pathway genes (Figure 14). The results showed that the levels of IP3R and AP-1 were significantly increased after treatment with PAMK compared with NC group, but the levels of CD28, PLCγ-1 and NFAT were similar to those of NC group. Additionally, siCD28 could significantly inhibit the mRNA expression of CD28, IP3R, PLCγ-1 and NFAT, but not AP-1. However, the PAMK + siCD28 group was significantly different from the siCD28 group and more similar to the NC group. All the results showed that PAMK could alleviate the decline in the mRNA expression levels of CD28, IP3R, PLCγ-1, NFAT, and AP-1 induced by siCD28.

## Discussion

CTX is often used to generate immunosuppressive models to research the immune function of various kinds of lymphocytes because it can abrogate the development and function of lymphocytes (Li et al., 2018; Wang et al., 2018; Han et al., 2019), causing special damage to humoral and cellular immunity (Guo et al., 2012a; Wang et al., 2012; Guo et al., 2019). It has been reported that CTX can induce a substantial reduction of mouse weight changes, spleen and thymus indexes, and induced natural killer (NK) cytotoxicity and proliferation activities of lymphocytes. It also downregulates the levels of IL-2, IL-6, TNF-α, and IFN-γ and causes an imbalance in CD4+/CD8+ (Wang et al., 2012; Wang et al., 2018; Ibrahim et al., 2019; Meng et al., 2019). Therefore, a mouse model of immunosuppression was constructed via the injection of CTX, and the morphology and death of spleen cells were measured. The results indicated that CTX could lead to spleen characterized by lymphocytes having an incomplete morphology, a loose and irregular arrangement, a large number of macrophages, fibrous tissue hyperplasia, increased apoptotic cells and apoptotic bodies, which indicated that the mouse immunosuppressive model was successful constructed. These phenomena could be explained by the damage caused by CTX to immune organs such as the spleen, the inhibition of the production of haematopoietic stem cells, and the induction of abnormal lymphocyte morphology and function (Li et al., 2018; Sun et al., 2018). Here, we found that PAMK could significantly increase the spleen index by improving the weight of the spleen in mice. The spleen cells in HE and TEM showed sparse and irregular, and had many apoptotic bodies in the CTX group, and these reasons may cause the decrease of spleen index in CTX group. When it comes to PAMK group, we found that the spleen index and the spleen cells shape have no significant difference with C group, which means that PAMK maintained the stability of cell morphology to protect the cells, tissues and organs. CTX could inhibite bone marrow hematopoietic function, and the stem cells in the spleen will have extramedullary hematopoietic function after being stimulated by CTX. Megakaryocyte precursor cells are typical cells of extramedullary hematopoiesis. When cellular immune response occurs, T cells in the periarterial lymphatic sheath divide and proliferate, and the sheath become thick. Thus, the thickness of periarterial lymphatic sheath significantly thinned and the number of megakaryocyte precursor cells significantly increased in CTX group when compared with C group. In our research, we found the PAMK + CTX group was significantly different from the CTX group, which indicated that PAMK might alleviate immunosuppressive mice by restoring the spleen cells morphology, increasing the thickness of the periarterial lymphatic sheath, alleviating the inhibition of bone marrow hematopoietic function caused by CTX and decreasing the death of spleen cells. These findings were consistent with previous research (Fu et al., 2018; Fan et al., 2019; Guo et al., 2019; Meng et al., 2019). But although all these results showed that PAMK could maintain the stability of the spleen morphology, we should test some immune factors to prove the immune level.

Cytokines play an important role in disease and health, especially in infection, inflammation, and cancer (Fu et al., 2018; Fan et al., 2019; Guo et al., 2019; Meng et al., 2019). Increased levels of IL-2, IL-6, TNF-α, and IFN-γ can enhance the activity of macrophages and improve specific immunity (Jin et al., 2017; Li et al., 2018; Hu et al., 2019; Liu et al., 2019). IL-6 could promote B lymphocytes proliferation and differentiation, and promote Th1/Th2 cell immune response. TNF-α could promote lymphocytes differentiation and improve the level of humoral and cellular immunity (Guo et al., 2019). However, in our research, the PAMK + CTX group showed that PAMK could not only significantly improve the level of IL-2, IL-6, TNF-α, and IFN-γ in serum but also increase expression of relative mRNA when compared with CTX group, which indicated that PAMK could improve immunity functions by improving the levels of immune cytokines. Interestingly, we found that the data of cytokines level in serum and mRNA level was different. The mRNA expression level in PAMK group increased more significantly and CTX group decreased more significantly when it compared with cytokines level in serum. This phenomenon might be caused by the fact that ELISA measured the content of cytokine secreted protein in serum while the level of the protein translation was determined by many other factors in body, so the level of cytokine protein secreted in serum is not as significant as the level of mRNA. Some researchers have also compared mice treated with *Grifola frondosa,* quinoa crude polysaccharides administration of *Lycium barbarum* polysaccharides and CTX, and showed that in the polysaccharide + CTX group, spleen and thymus indexes were significantly increased and immunoglobulin M (IgM), IFN-γ, IL-2, IL-6, TNF-α levels effectively increased when it compared with CTX group (Ding et al., 2019b; Guo et al., 2019). In some researches we also found that polysaccharides have more significant transcription than translation levels in increasing the expression level of cytokines (Fei et al., 2019). These results were similar to those of our study. Taken together, these findings confirm that PAMK can alleviate the decline level of cytokines after inhibited by CTX.

Th1 mainly secretes IL-2, IL-6, IFN-α, IFN-γ, TNF-α and tumor necrosis factor *β* (TNF-β) (Cao et al., 2019). The cytokines could mediate the immune response related to cell and local inflammation by assisting in the production of antibodies, increasing the activation of macrophages and removing intracellular pathogens. However, Th2 secrete IL-4, IL-5, IL-10 (Zhang et al., 2017; Zhou et al., 2018), which could inhibit the activation of Th1 lymphocytes. When the body is in a healthy state, Th1/Th2 is in a dynamic equilibrium. When the cytokine types in the microenvironment change, the function of one subgroup of Th1 or Th2 will increase and that of the other subgroup will decrease, leading to the drift of Th1/Th2. In our research, we found that CTX could decline the level of Th1, increase the level of Th2, destroy the immune mechanism, make the balance of Th1/Th2 disrupted and decrease the ratio of Th1/Th2. However, PAMK + CTX group showed that PAMK markedly enhanced the ratio of Th1/Th2 in spleen T lymphocytes, which indicated that PAMK could affect the typing of T lymphocytes and promote T lymphocytes shift to Th1, making Th1/Th2 ratio nearly normal. PAMK could increase the level of Th1, so the cytokines secreted by Th1 in PAMK + CTX group, such as IL-2, IL-6, TNF-α, and IFN-γ were significantly increased when it compared with the CTX group. The results were consistent with our previous cytokine mRNA and ELISA detection analyses. Some researches also suggested that polysaccharide could improve the expression level of Th1 and maintain the T cell balance in mice (Cao et al., 2019; Deng et al., 2019; Zhao et al., 2019), which were similar to our research. In our research, we also found that PAMK could increase an important cytokines, IL-2, which could promote T cell differentiation and proliferation in each subgroup. So we speculate that the PAMK may play an important role in T lymphocytes activation.

T lymphocytes are important immune cells in body and participate in cellular immunity. The important hallmark of activation is the activation of the early and late activators CD25, CD69, and CD71 (Motamedi et al., 2016). In our research, we found that the levels of activated factors in PAMK + CTX was significantly increased when it compared with CTX, which suggested that PAMK could activate T lymphocytes. The results indicated that the mRNA level of CD28, PLCγ-1, IP3R, NFAT, and AP-1 in PAMK + CTX group were positively regulated in spleen and lymphocytes when compared with CTX group. These findings have important implications for understanding the molecular mechanisms of PAMK. Thus, the results suggested that PAMK could activate T lymphocytes by upregulating CD28/IP3R/PLCγ-1/AP-1/NFAT signal pathway. It also could through the IL-2 released to promote each subgroup T cell differentiation and proliferation to induce the production of various cytokines thereby alleviate the immunosuppression caused by CTX. The polysaccharide-protein complex from *Lycium barbarum* L. has been reported to induce secretion of IL-2 and IFN-γ in T lymphocytes via promoting the transcriptional activities of NFAT and AP-1 (Chen et al., 2008; Motamedi et al., 2016) Conversely, both gossypol and pseudoephedrine were shown to suppress mouse T lymphocytes via inhibition of NF-κB, NFTA and AP-1 (Chen et al., 2008; Fiebich et al., 2012; Song et al., 2013). Some studies have suggested that AP-1, as a cooperative transcription factor, is more beneficial to the IL-2-based NFAT activation system (Chen et al., 2008; Zhang et al., 2018; Zhang et al., 2019). In this study, it was found that PAMK could increase the gene levels of AP-1 and NFAT in immunosuppressed mice induced by CTX, suggesting that PAMK might play a synergistic role through AP-1 and NFAT.

In conclusion, PAMK could activate T lymphocytes through CD28/IP3R/PLCγ-1/AP-1/NFAT signal pathway, regulate the Th1/Th2 balance, promote the proliferation and differentiation of T lymphocytes, promote the secretion of IL-2, IL-6, TNF-α, and IFN-γ, restore the spleen cells morphology, reduce dead spleen cells to alleviate the immune suppression caused by CTX thereby restore the organism back to the normal immunity level.

## Date Availability Statement

All datasets generated for this study are included in the manuscript/supplementary material.

## Ethics Statement

The animal study was reviewed and approved by Animal Ethics Committee of Zhongkai College of Agricultural Engineering.

## Author Contributions

WL contributed to the hypothesis generation, experimental design, data interpretation, and manuscript preparation. XX and DX conducted the experiments. YT and NC contributed to the data interpretation.

## Funding

This work was supported by Basic and Applied Basic Research Fund Project of Guangdong Province (2019A1515110106); Science and Technology Plan Project of Guangzhou (grant No.201904010076); Innovation fund support of Zhongkai College of Agricultural Engineering (grant No.2019A04). This funding had no impact on our study design or collection, analysis, and interpretation of the data. Further support was provided solely from institutional and/or departmental sources.

## Conflict of Interest

The authors declare that the research was conducted in the absence of any commercial or financial relationships that could be construed as a potential conflict of interest.
