# Supplementary material for: Polysaccharide of Atractylodes macrocephala Koidz (PAMK) Alleviates Cyclophosphamide-induced Immunosuppression in Mice by Upregulating CD28/IP3R/PLCγ-1/AP-1/NFAT Signal Pathway
Source: Front Pharmacol. 2020 Dec 8;11:529657. doi: 10.3389/fphar.2020.529657 (PMC7753208; doi:10.3389/fphar.2020.529657)
Supplement: Supplementary file 1 [file datasheet1.zip › WB/Lymphocytes WB/explain.docx]

All (1) pictures contain 6 samples, 1-1~1-6 represent group NC, 2-1~2-6 represent group PAMK, 3-1~3-6 represent group siCD28, 4-1~4-6 represent group PAMK+siCD28.

All (2) pictures contain another 3 samples which is different form pictuer (1), 1-2, 1-3, 1-4 represent group NC, 2-1, 2-2, 2-3 represent group PAMK, 3-1, 3-3, 3-4 represent group siCD28, 4-2, 4-3, 4-4 represent group PAMK+siCD28.

Protein size:CD28 47 KDa

IP3R 222KDa

NFAT 150KDa

PLCγ-1 146KDa

GAPDH 36KDa

AP-1 48KDa
