# Supplementary material for: Polysaccharide of Atractylodes macrocephala Koidz (PAMK) Alleviates Cyclophosphamide-induced Immunosuppression in Mice by Upregulating CD28/IP3R/PLCγ-1/AP-1/NFAT Signal Pathway
Source: Front Pharmacol. 2020 Dec 8;11:529657. doi: 10.3389/fphar.2020.529657 (PMC7753208; doi:10.3389/fphar.2020.529657)
Supplement: Supplementary file 1 [file datasheet1.zip › WB/spleen WB/explain.docx]

All pictures contain 3 samples, 1-1004, 1-1005, 1-1007 represent group C, 3-1024, 3-1027, 3-1028 represent group PAMK, 5-1035, 5-1050, 5-1054 represent group CTX, 4-2, 4-3, 4-4 represent group PAMK+CTX.

Protein size:CD28 47 KDa

IP3R 222KDa

NFAT 100KDa

PLCγ-1 146KDa

GAPDH 36KDa

AP-1 48KDa
