# Supplementary figures and images for: Polysaccharide of Atractylodes macrocephala Koidz (PAMK) Alleviates Cyclophosphamide-induced Immunosuppression in Mice by Upregulating CD28/IP3R/PLCγ-1/AP-1/NFAT Signal Pathway
Source: Front Pharmacol. 2020 Dec 8;11:529657. doi: 10.3389/fphar.2020.529657 (PMC7753208; doi:10.3389/fphar.2020.529657)

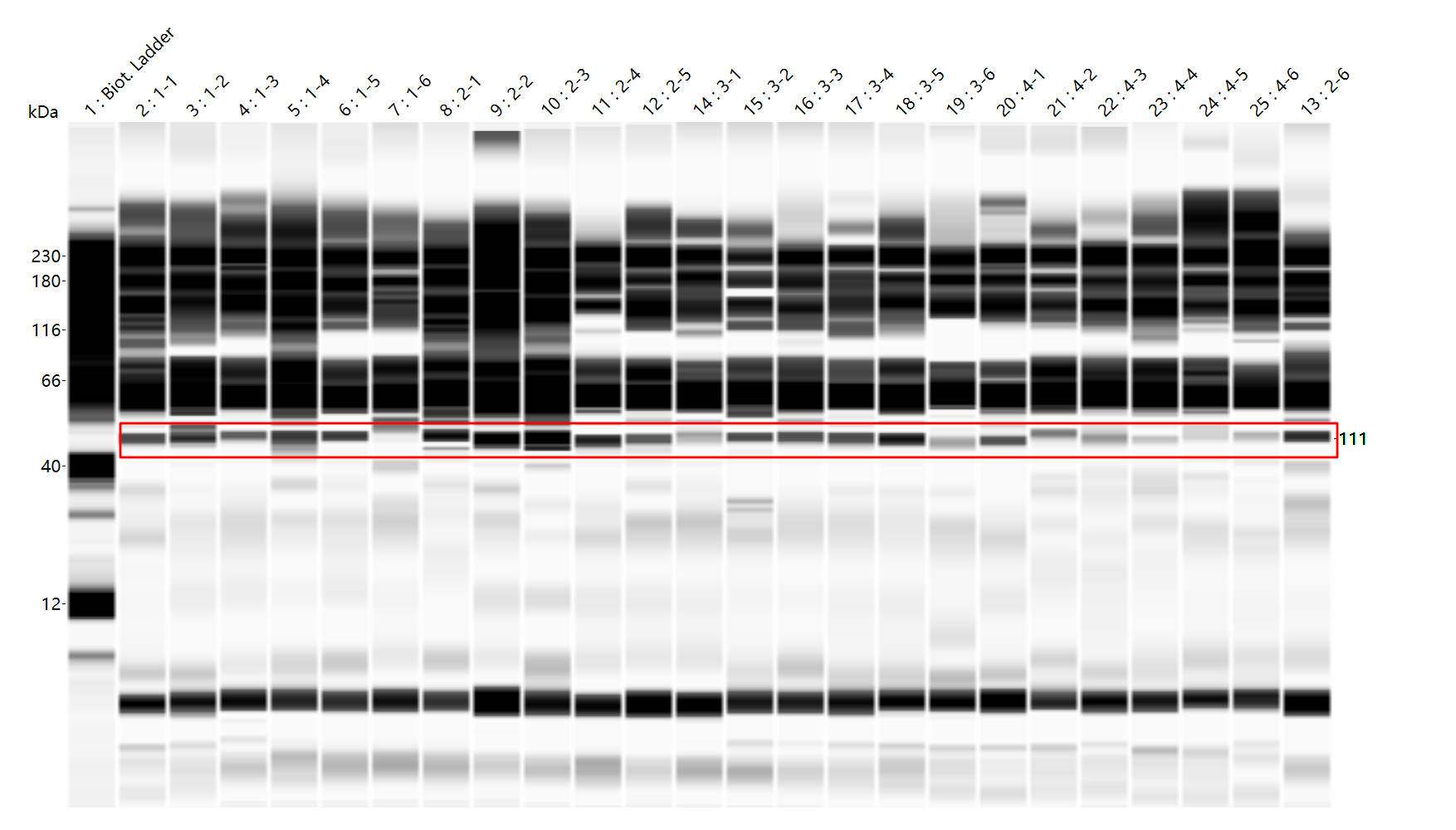

Supplement: Supplementary file 1 [file datasheet1.zip › WB/Lymphocytes WB/AP-1(1).png]

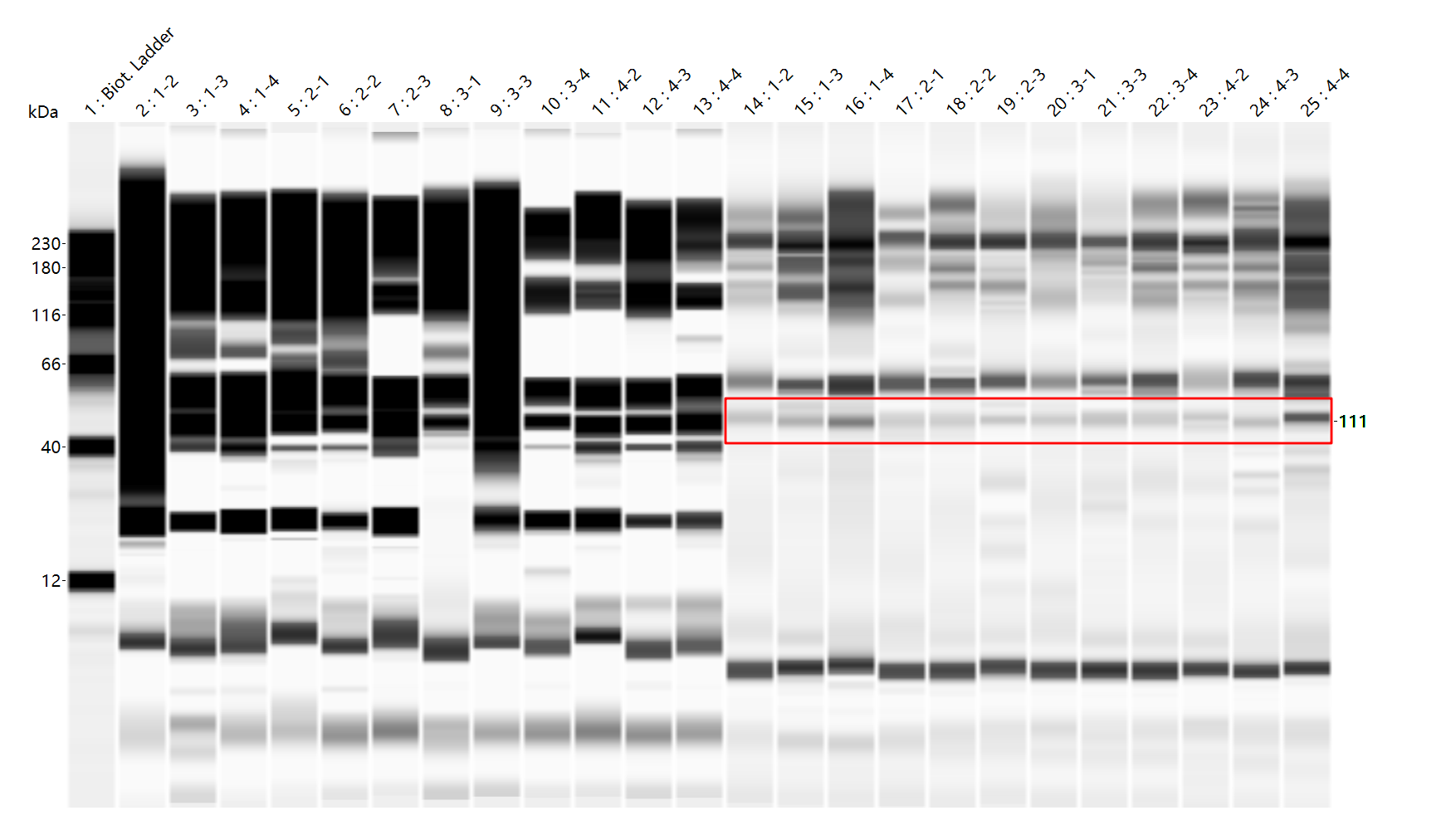

Supplement: Supplementary file 1 [file datasheet1.zip › WB/Lymphocytes WB/AP-1(2).png]

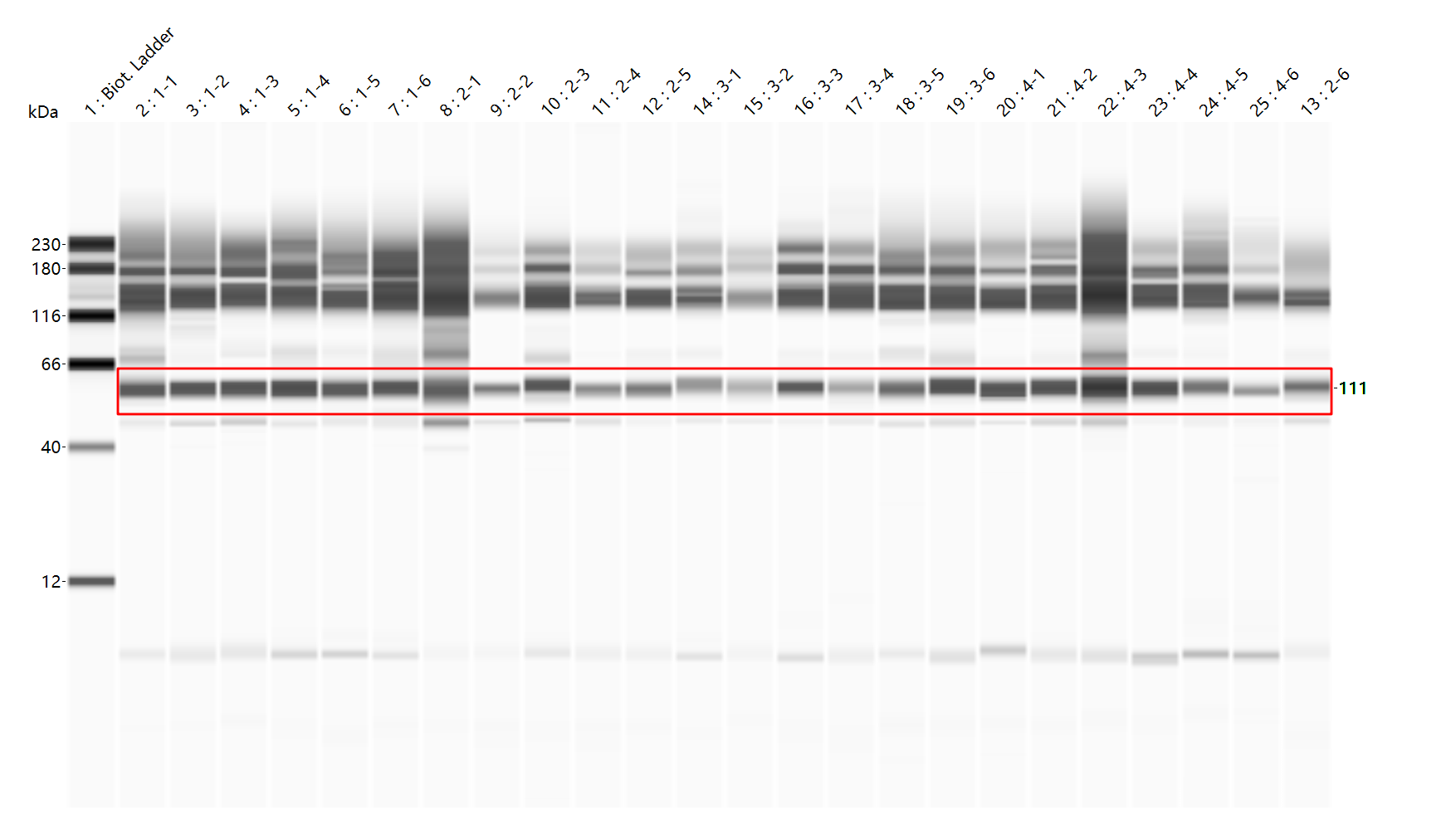

Supplement: Supplementary file 1 [file datasheet1.zip › WB/Lymphocytes WB/CD28 (1).png]

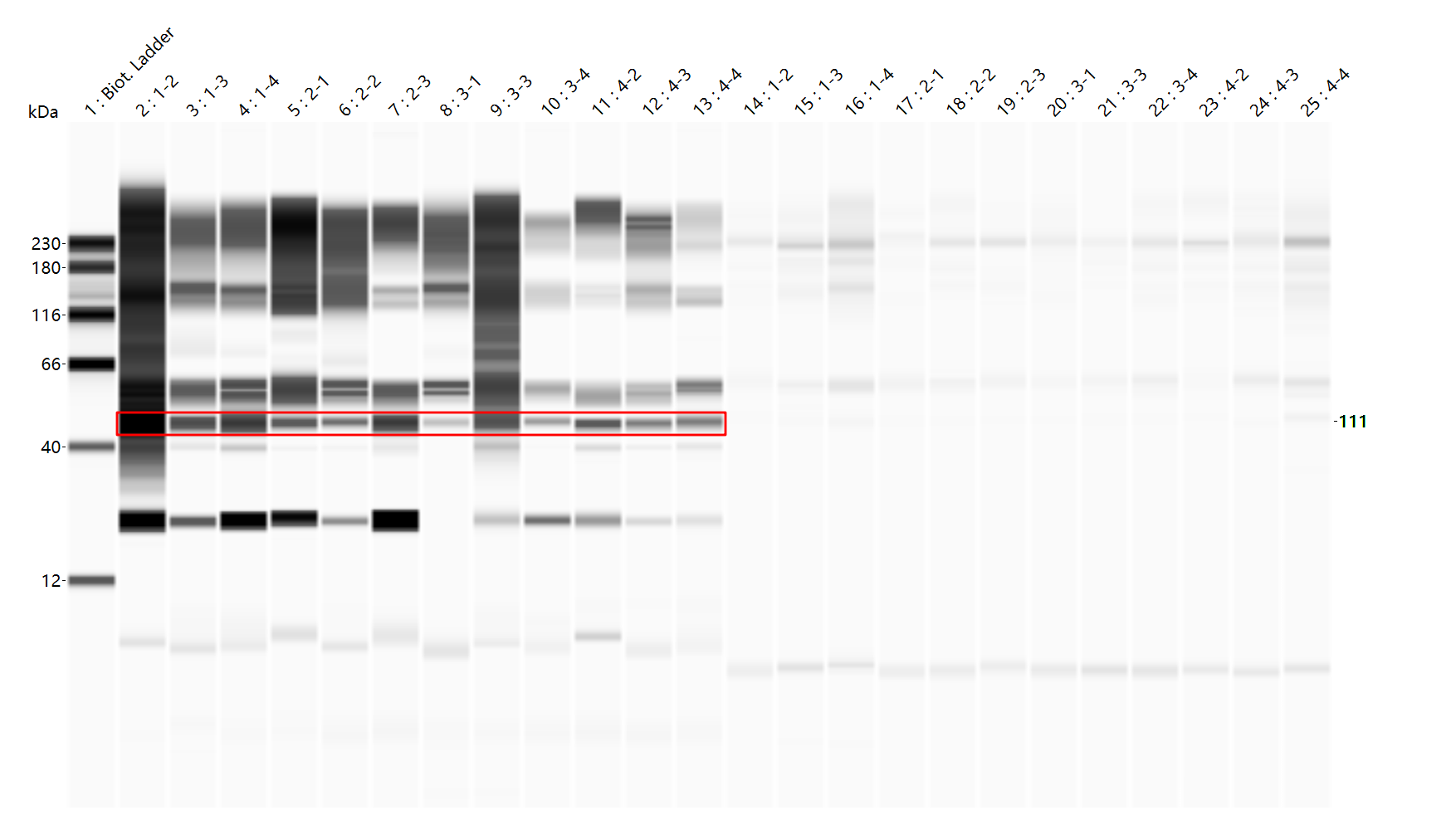

Supplement: Supplementary file 1 [file datasheet1.zip › WB/Lymphocytes WB/CD28(2).png]

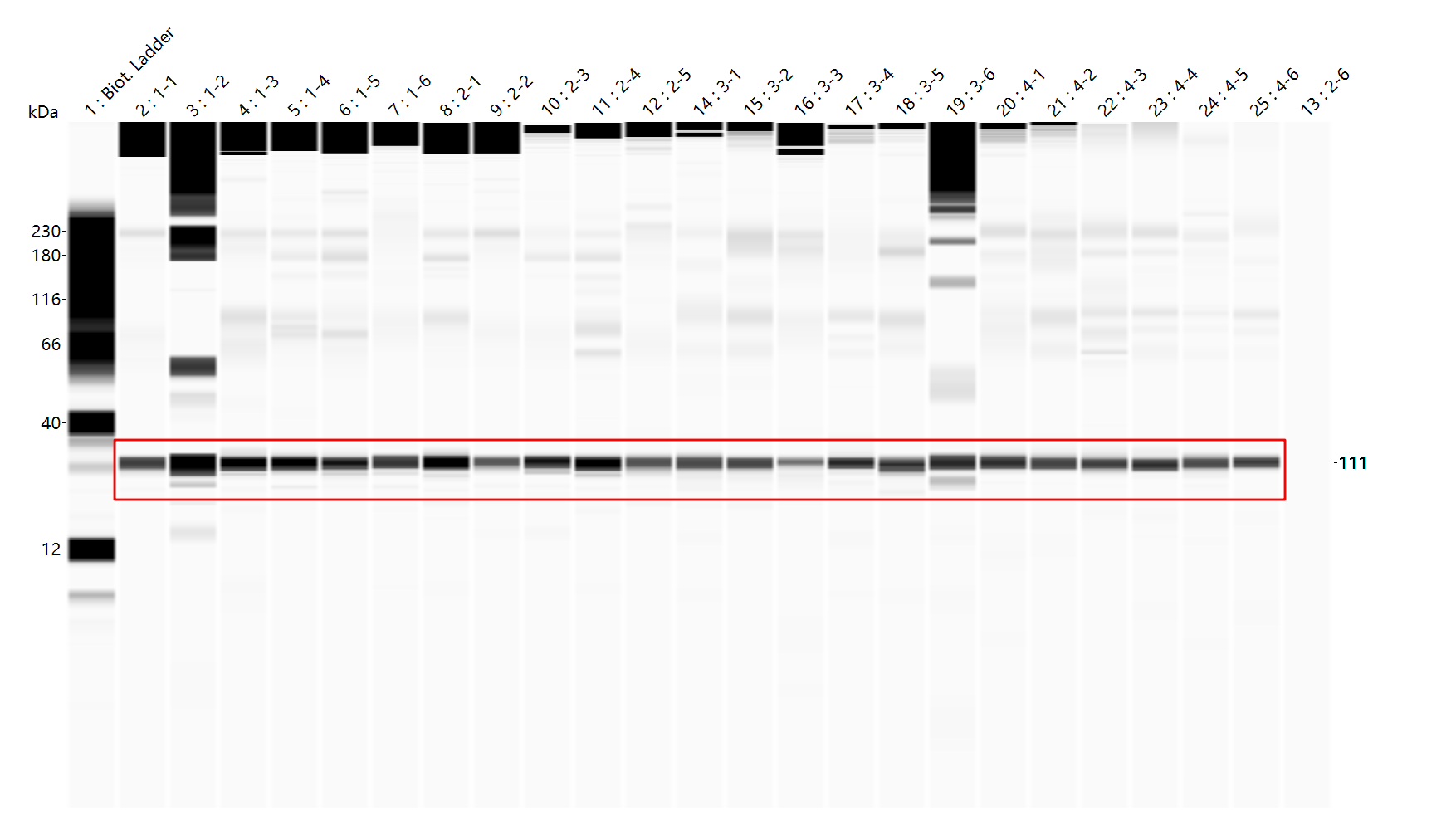

Supplement: Supplementary file 1 [file datasheet1.zip › WB/Lymphocytes WB/GAPDH (1).png]

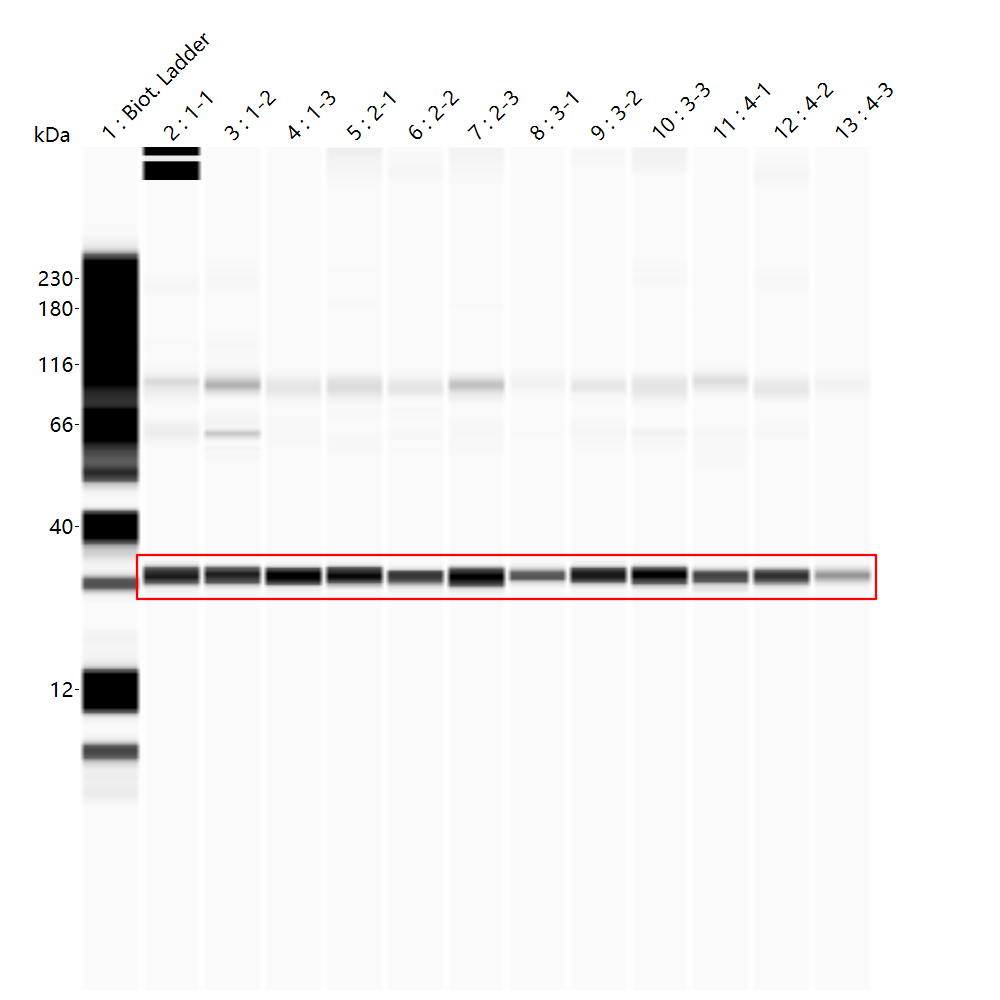

Supplement: Supplementary file 1 [file datasheet1.zip › WB/Lymphocytes WB/GAPDH(2).png]

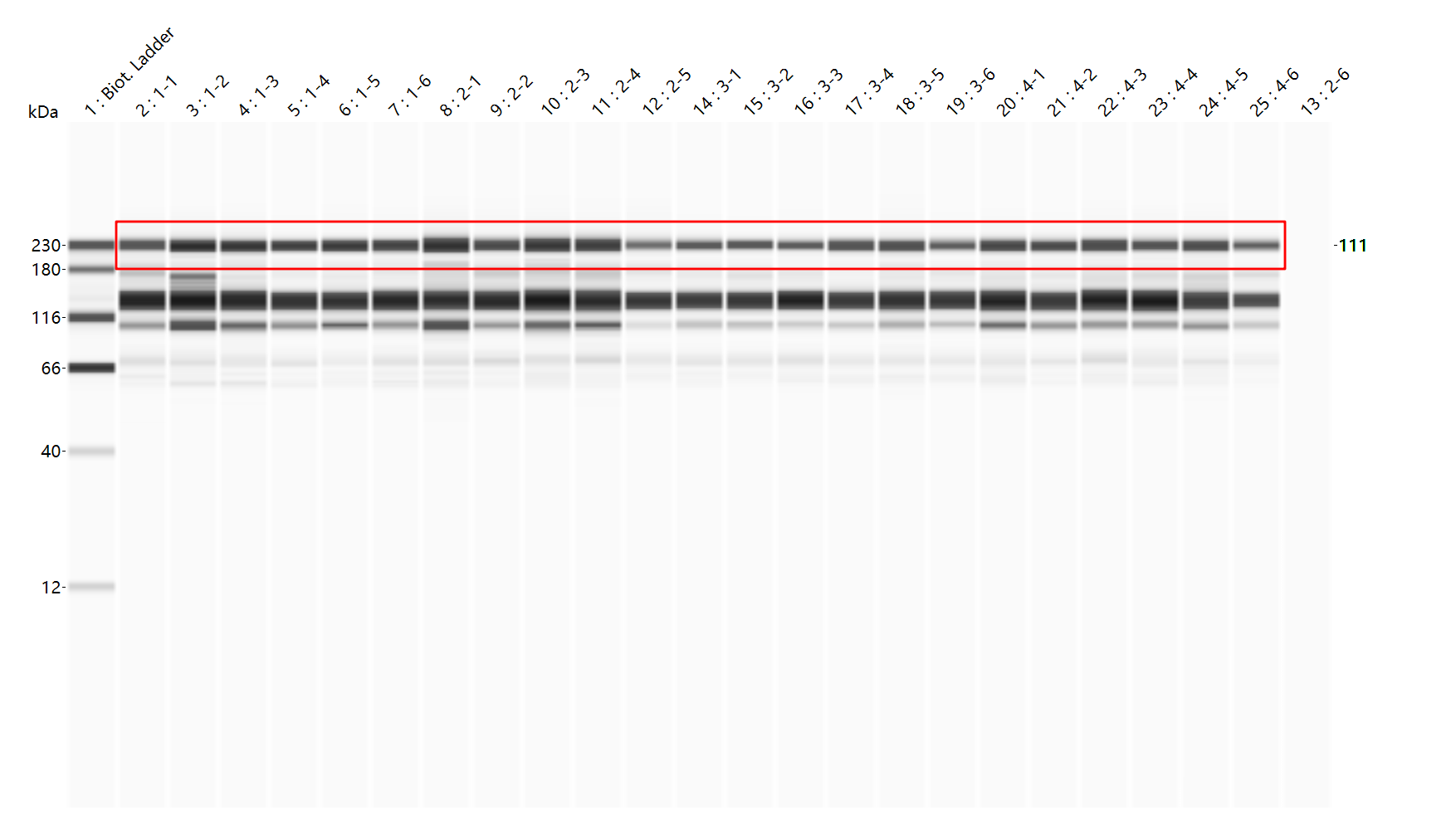

Supplement: Supplementary file 1 [file datasheet1.zip › WB/Lymphocytes WB/IP3R(1).png]

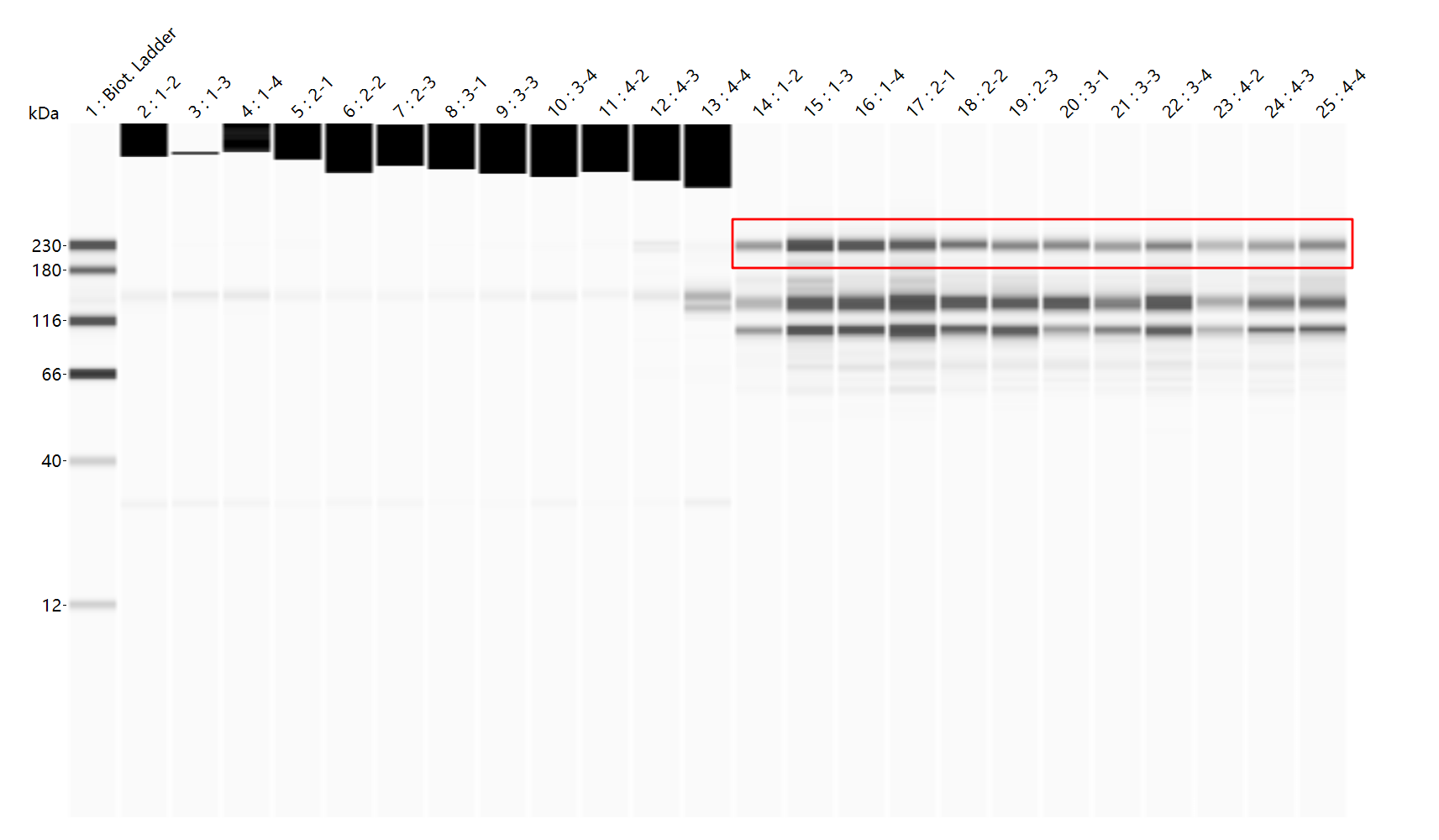

Supplement: Supplementary file 1 [file datasheet1.zip › WB/Lymphocytes WB/IP3R(2).png]

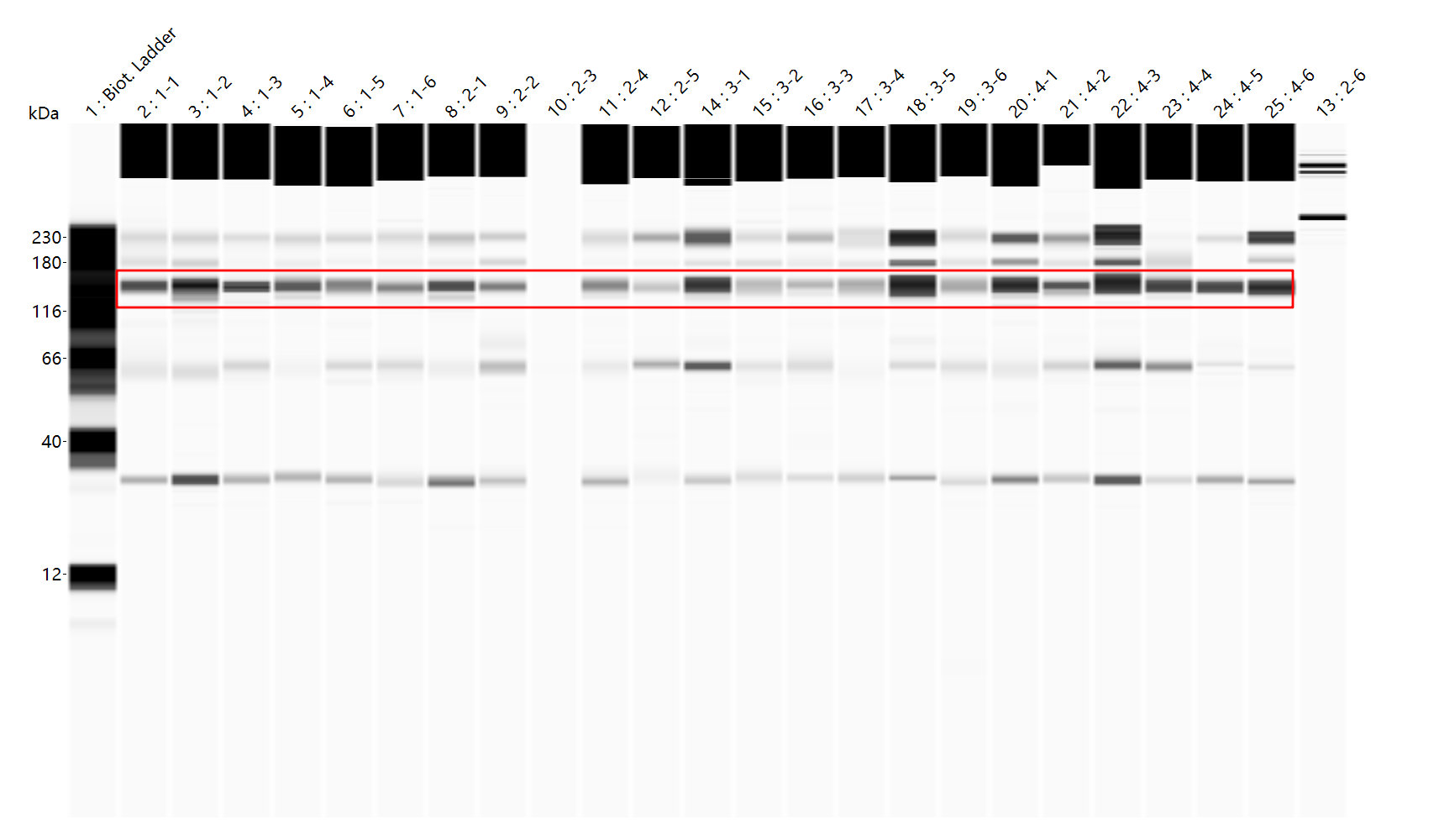

Supplement: Supplementary file 1 [file datasheet1.zip › WB/Lymphocytes WB/NFAT(1).png]

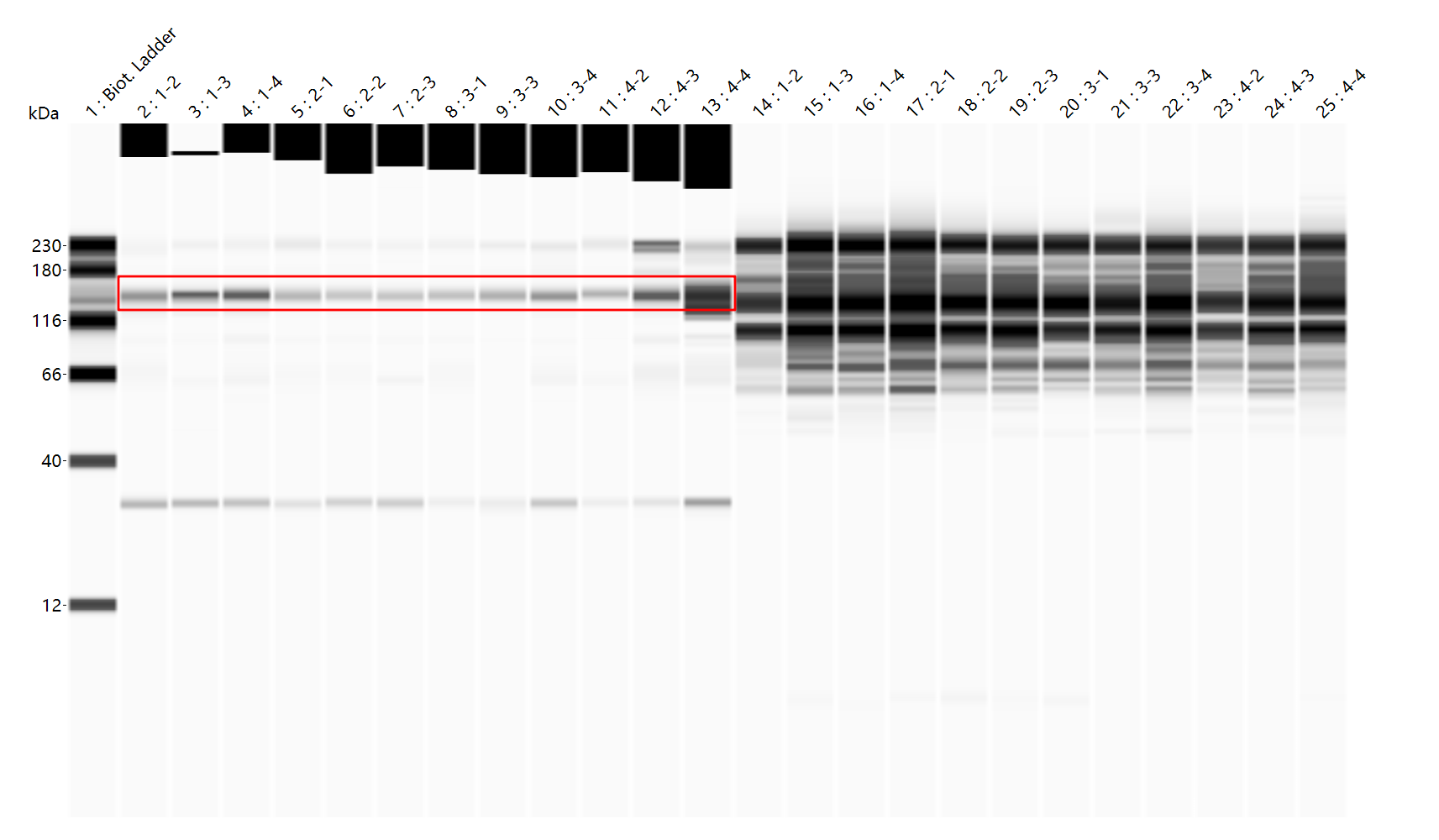

Supplement: Supplementary file 1 [file datasheet1.zip › WB/Lymphocytes WB/NFAT(2).png]

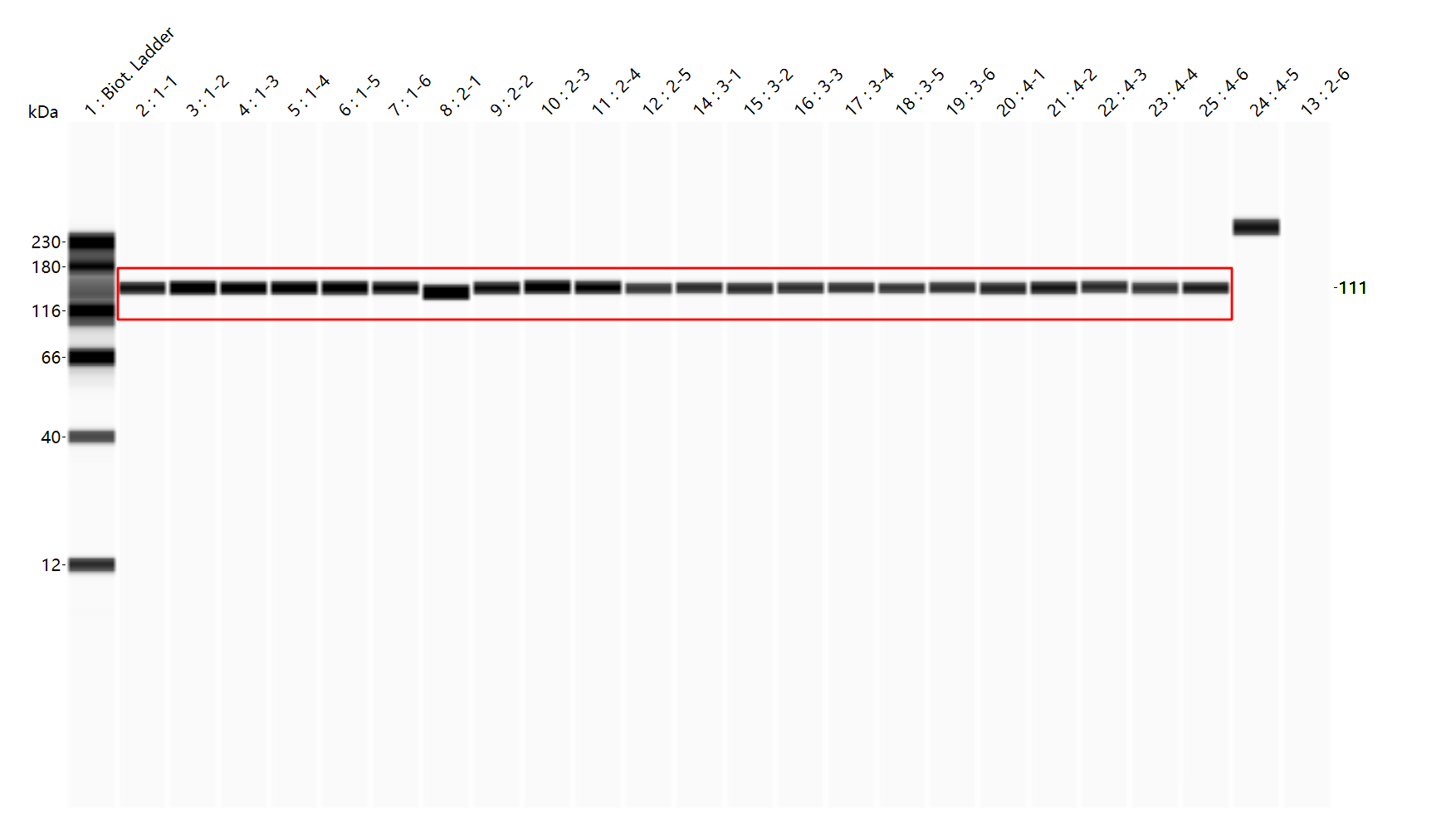

Supplement: Supplementary file 1 [file datasheet1.zip › WB/Lymphocytes WB/PLCr-1(1).png]

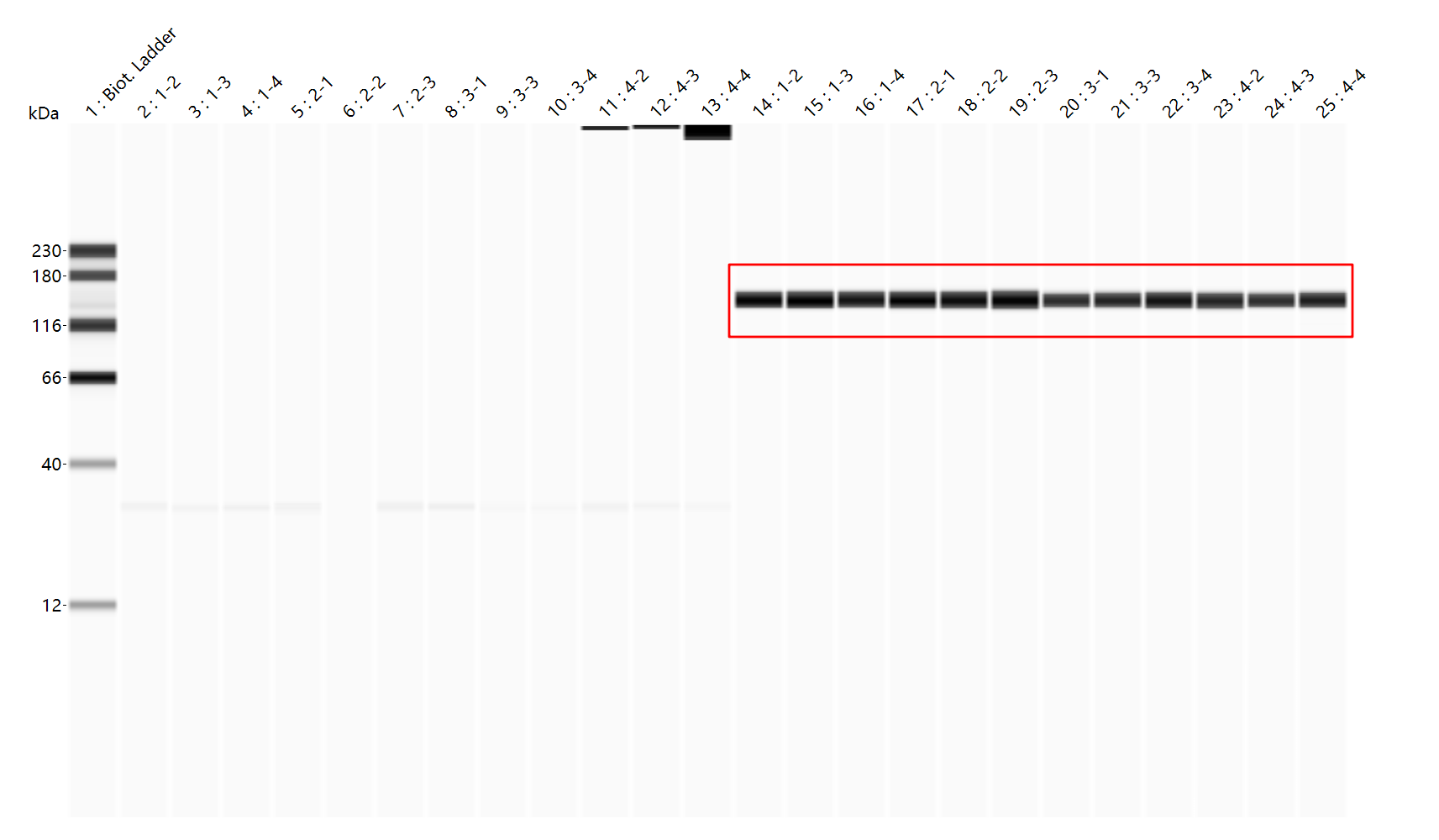

Supplement: Supplementary file 1 [file datasheet1.zip › WB/Lymphocytes WB/PLCr-1(2).png]

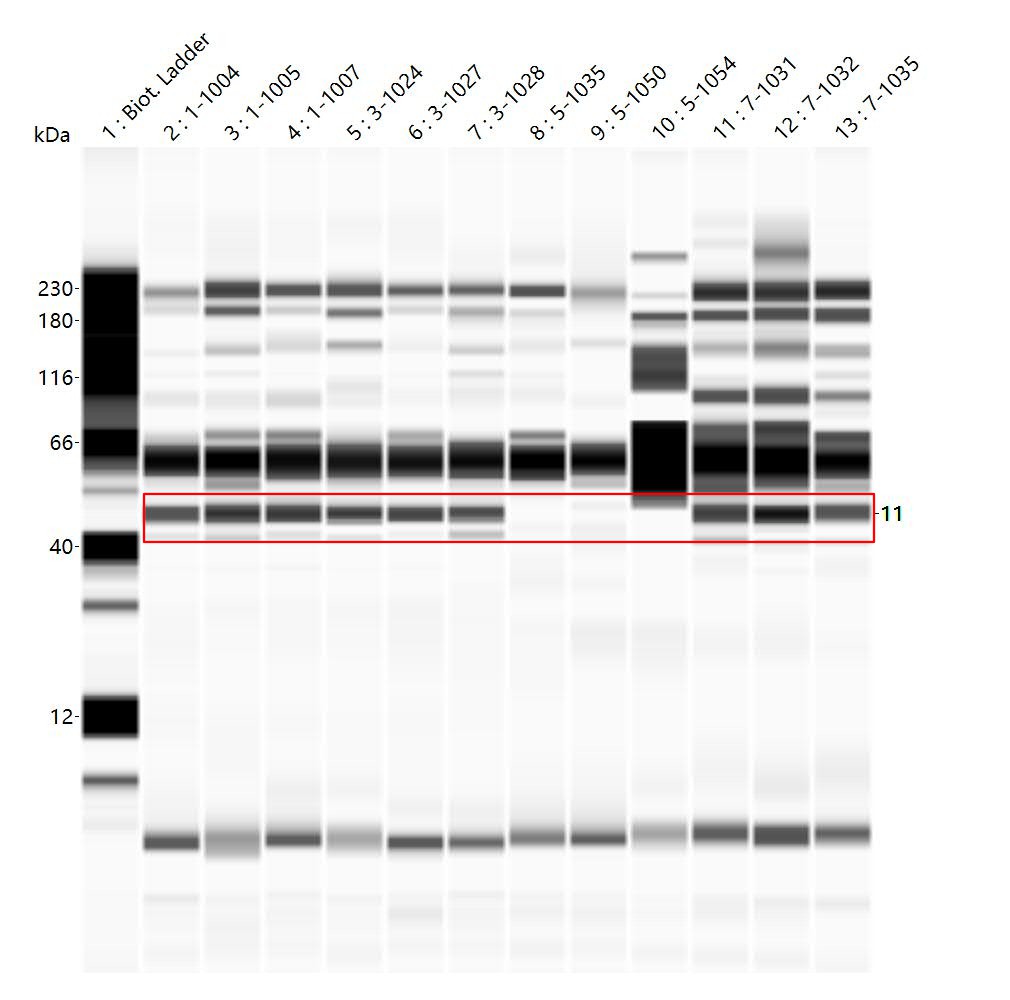

Supplement: Supplementary file 1 [file datasheet1.zip › WB/spleen WB/AP-1.jpg]

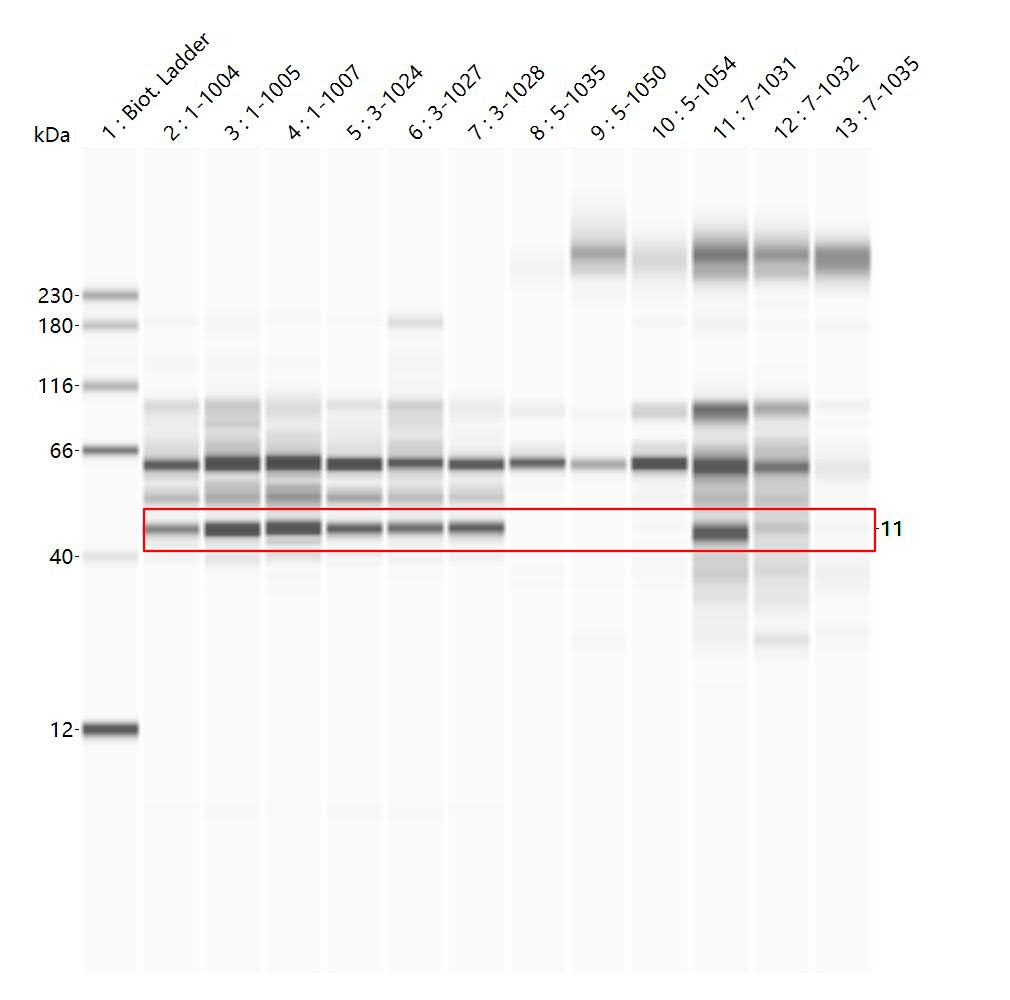

Supplement: Supplementary file 1 [file datasheet1.zip › WB/spleen WB/CD28.jpg]

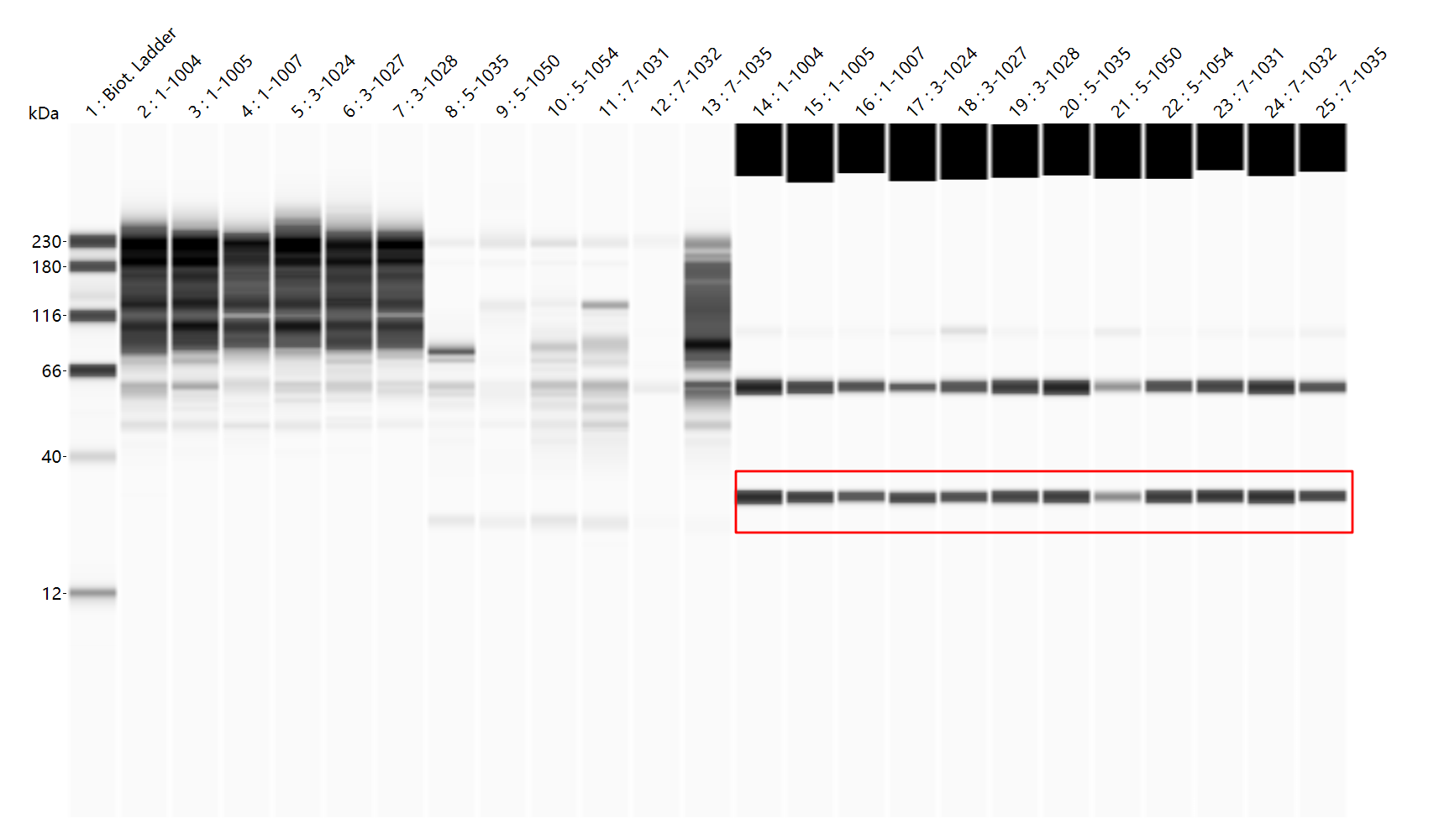

Supplement: Supplementary file 1 [file datasheet1.zip › WB/spleen WB/GAPDH.png]

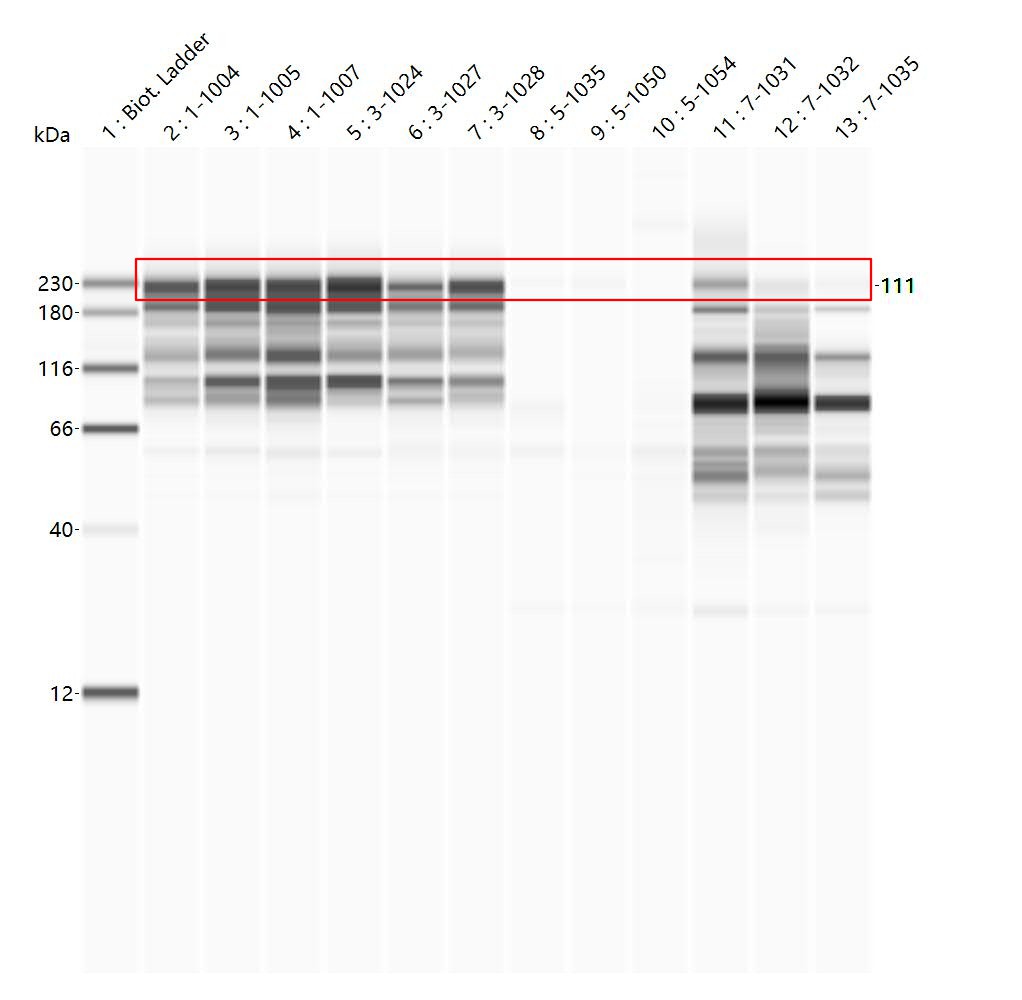

Supplement: Supplementary file 1 [file datasheet1.zip › WB/spleen WB/IP3R.jpg]

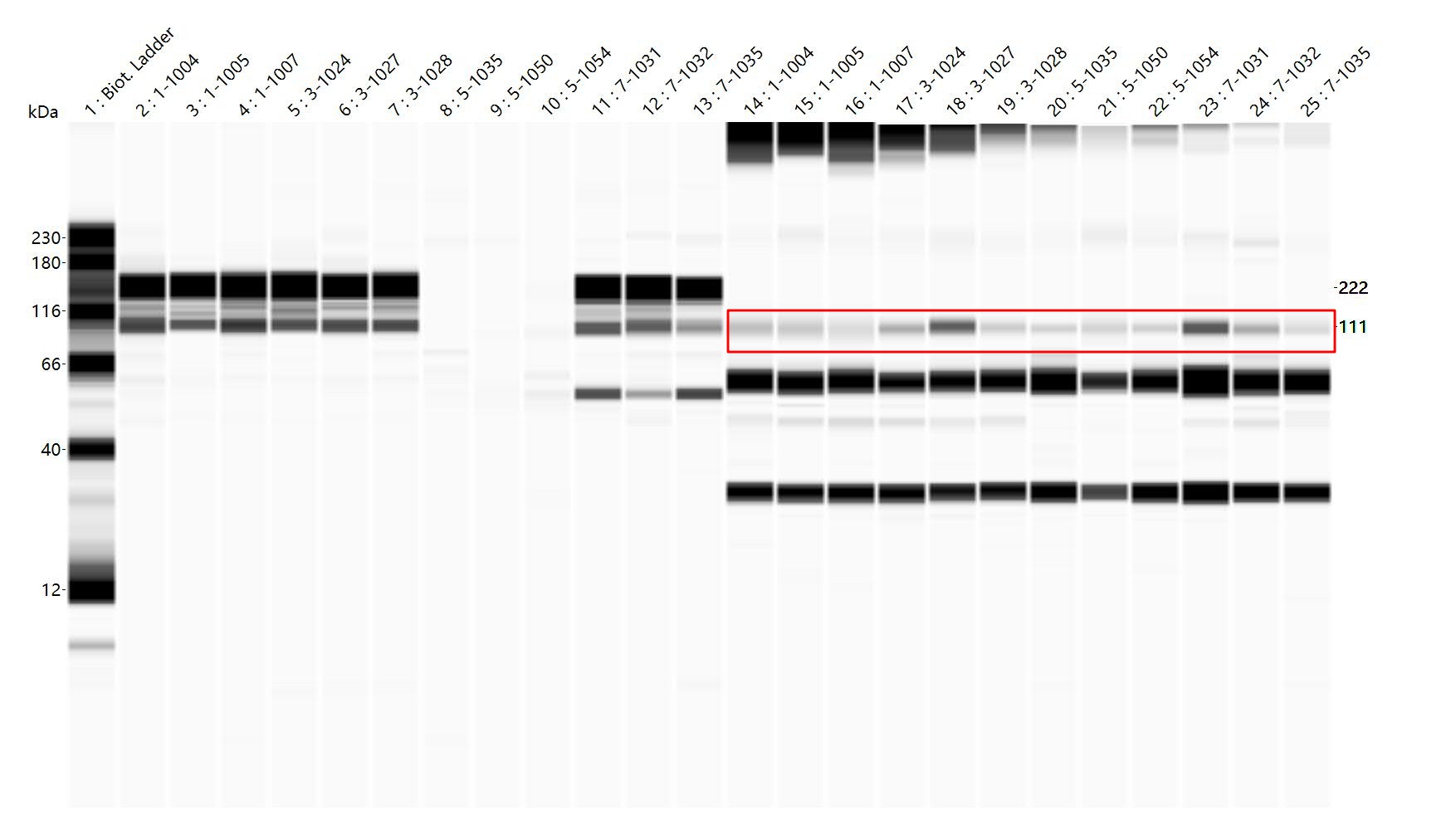

Supplement: Supplementary file 1 [file datasheet1.zip › WB/spleen WB/NFAT.jpg]

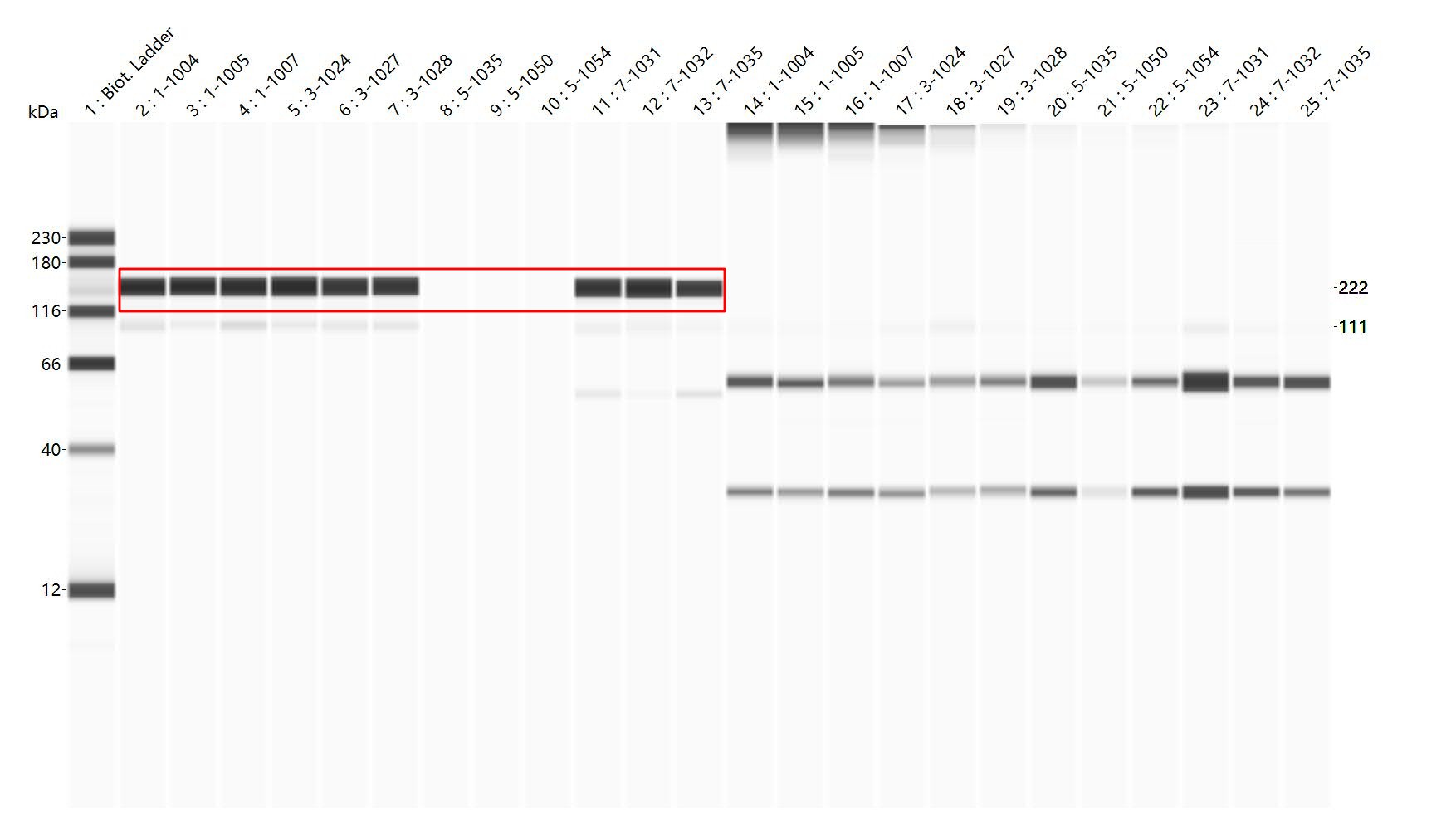

Supplement: Supplementary file 1 [file datasheet1.zip › WB/spleen WB/PLCr-1.jpg]
